# Architecting Anode Current Collectors for Practical Anode-Free Alkali Metal Batteries

**DOI:** 10.1007/s40820-026-02355-w

**Published:** 2026-10-12

**Authors:** Kai Tang, Zihan Shen, Wen Xie, Pengfei Song, Longcheng Zhang, Dongshuang Wu, Zhichuan J. Xu

**Affiliations:** School of Materials Science and Engineering, Nanyang Technological University, 50 Nanyang Avenue, Singapore, 639798 Singapore

**Keywords:** Anode-free lithium metal battery, Anode-free sodium metal battery, Anode-free potassium metal battery, Current collector engineering, Solid electrolyte interphase

## Abstract

Anode-free alkali metal batteries (AFAMBs) are emerging as a compelling strategy to surpass the cell-level energy density limits of Li-ion batteries while simplifying battery configuration. In these systems, the working anode forms in situ on a bare current collector and is continuously reshaped during cycling with a limited alkali metal inventory. As a result, the anode current collector is not merely a passive electron conductor, but a multifunctional interfacial platform that governs alkali metal nucleation, growth, stripping, and interphase evolution. This review examines the fundamental differences among anode-free lithium, sodium, and potassium systems and highlights key performance metrics for practical AFAMBs. Recent current collector engineering strategies developed for these chemistries are then summarized. Essential parameters shaping current collector design are identified, including electronic conductivity, alkali metal affinity, collector morphology, and interphase stability. Finally, practical design principles are presented to balance electrochemical performance with manufacturability under realistic operating conditions. This review aims to clarify the alkali metal-dependent design logic of current collectors and to provide guidance for the practical development of next-generation anode-free batteries.

## Highlights

- A systematic comparison of anode-free lithium, sodium, and potassium battery systems, emphasizing their distinct energy density, volume change, and interfacial instability.
- A current-collector-centered analysis of anode-free alkali metal batteries, highlighting how current collectors govern alkali metal nucleation, growth, stripping, and interphase evolution under limited alkali metal inventory.
- A practical framework for the development of anode-free alkali metal batteries, integrating performance metrics, current collector design parameters, and manufacturability constraints.

## Graphical abstract

**Figure:**
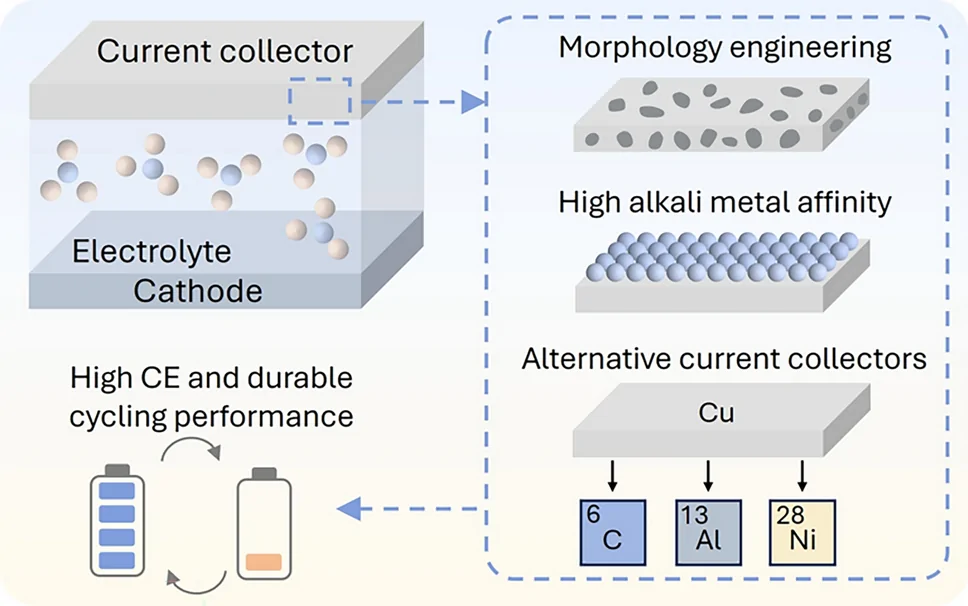

## Introduction

Rechargeable lithium-ion batteries (LIBs) underpin modern portable electronics and electric vehicles, but their practical energy density and cost are increasingly constrained by intercalation-based electrodes and inactive cell components [1–4]. These limitations have renewed interest in batteries that employ metallic alkali anodes, including lithium, sodium, and potassium, as negative electrodes with high specific capacities and low redox potentials [5–8]. Within this broad family, anode-free alkali metal batteries (AFAMBs) have emerged as a conceptually simple yet powerful strategy to exploit alkali metal without assembling thick excess metal foils.

In an anode-free configuration, the anode side initially contains only a bare current collector, with no pre-deposited alkali metal [9–13]. During the first charge, active alkali metal is plated onto the anode current collector to form the working anode. By eliminating thick excess metal foil, this configuration can substantially increase gravimetric and volumetric energy densities and avoid the direct handling of highly reactive alkali metals. In recent years, anode-free lithium metal batteries (AFLMBs) have attracted intense attention as promising candidates for next-generation energy storage, with systematic evaluations of their theoretical energy limits, failure modes, and design space [14–17]. Building on these advances, anode-free concepts have more recently been extended to anode-free sodium metal batteries (AFSMBs) and anode-free potassium metal batteries (AFPMBs) [18–20].

Nevertheless, AFAMBs face several fundamental and engineering challenges that are distinct from those in excess metal anode cells and are often more severe [21–25]. Because the entire active inventory resides in the cathode at assembly, even modest irreversibility during plating and stripping rapidly leads to early capacity loss. Furthermore, the working anode forms dynamically during the first charge and is continuously reshaped during cycling. Its morphology is therefore highly sensitive to the current collector structure, interphase chemistry, stack pressure, and the formation protocol. Inhomogeneous nucleation and growth can generate porous deposits, electrically isolated dead metal, and an unstable solid electrolyte interphase (SEI), which promote interfacial contact loss and short-circuit risk [26, 27].

Among the various strategies proposed to address these challenges, current collector engineering has emerged as one of the most direct and versatile approaches. In AFAMBs, the anode current collector is a decisive interfacial platform that governs alkali metal deposition behavior and interphase evolution under limited metal inventory. Its composition, surface chemistry, electronic structure, and morphology can strongly influence whether the deposited alkali metal remains dense, well adhered, and electrochemically accessible throughout cycling. Accordingly, current collector design plays a central role in translating the conceptual advantages of AFAMBs into practically viable batteries.

This review frames AFAMBs from the current collector viewpoint, highlighting how current collector design stabilizes interphase chemistry and conserves the limited alkali metal inventory. We first compare the intrinsic differences among the three AFAMB systems and discuss the important performance metrics for anode-free battery evaluation. Subsequent sections summarize several representative current collector designs for anode-free lithium, sodium, and potassium systems. Key parameters governing alkali metal deposition on bare current collectors are then discussed, including electronic conductivity, alkali metal affinity, collector morphology, and interphase stability. Collectively, this review highlights cross-cutting concepts and design principles for practical AFAMBs.

## From Li to Na and K: Expanding the Scope of Anode-Free Batteries

**Fig. 1:**
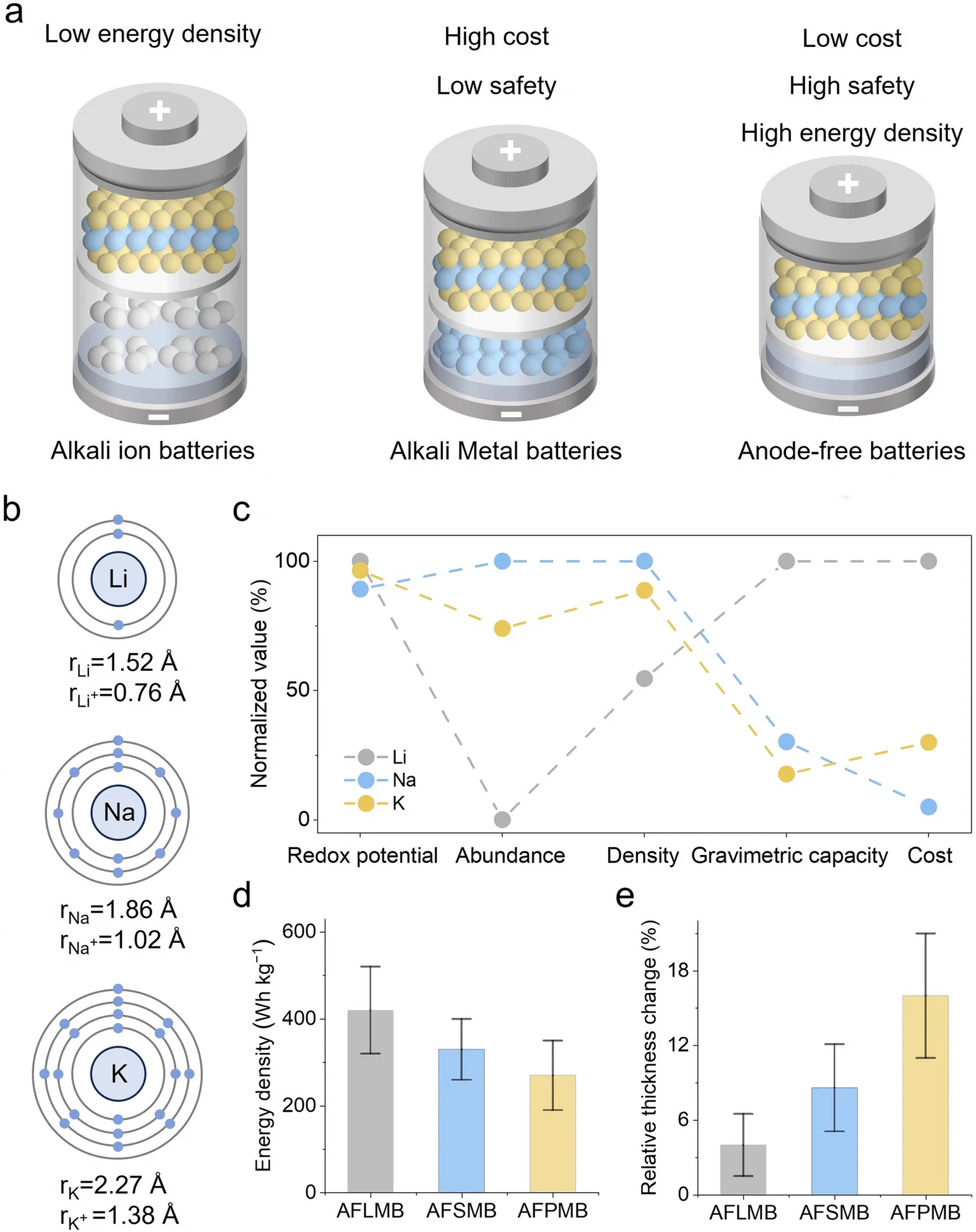
Different anode-free configurations and fundamental differences among Li, Na, and K. **a** Comparative schematic of structure and properties of alkali ion batteries, alkali metal batteries, and anode-free batteries. **b** Comparison of the atomic and ionic sizes of Li, Na, and K. **c** Normalized comparison of redox potential, abundance, density, gravimetric capacity, and cost for Li, Na, and K. **d** Energy-density comparison of AFLMB, AFSMB, and AFPMB. **e** Relative thickness change of AFLMB, AFSMB, and AFPMB after alkali metal plating

AFAMBs aim to simultaneously improve cell-level energy density, simplify cell configuration, and reduce manufacturing cost (Fig. 1a). The anode-free concept was initially explored primarily in Li-based systems, largely because Li possesses the most negative redox potential and the highest specific capacity among the alkali metals, making AFLMBs the most attractive route toward maximizing energy density [28–30]. In addition, the mature knowledge base and industrial infrastructure inherited from LIBs have further accelerated the development of anode-free Li systems. More recently, the scope of anode-free batteries has gradually extended to Na and K chemistries, driven by increasing interest in low-cost and sustainable large-scale energy storage.

Despite their shared anode-free configuration, Li, Na, and K differ markedly in their physicochemical characteristics and therefore in their practical design priorities [31–33]. The three alkali metals possess distinct atomic and ionic sizes (Fig. 1b), which strongly influence their solvation structures, ion migration, and metal deposition behavior. Li offers the highest gravimetric energy density and benefits from the most established battery manufacturing ecosystem, but concerns over cost and resource availability remain (Fig. 1c). Na is far more abundant and economically attractive, making AFSMBs particularly promising for large-scale storage, although the larger ionic radius and more sluggish interfacial kinetics of Na generally increase the difficulty of achieving dense and reversible metal deposition. Potassium is also highly abundant, but metallic K is not necessarily low cost in practice because its industrial production and market demand remain limited. Meanwhile, its larger ionic size, lower volumetric capacity, and higher chemical reactivity usually lead to more severe interfacial instability and poorer cycling reversibility, which presently hinder the development of anode-free potassium systems.

These intrinsic differences further translate into substantial variations in the battery-level characteristics of the corresponding AFAMB systems [34–36]. Owing to the much higher gravimetric capacity, volumetric capacity, and more negative redox potential of Li metal, AFLMBs generally deliver the highest energy density, with representative reports and practical targets commonly falling in the range of roughly 320–520 Wh kg^−1^ (Fig. 1d). In comparison, AFSMBs are typically discussed in the range of about 260–400 Wh kg^−1^, whereas reported AFPMBs remain much more limited and generally lower in practical energy density.

Moreover, these systems also exhibit markedly different volume changes during metal plating and stripping. At an equal areal capacity of 1 mAh cm^−2^, the theoretical plated metal thickness is about 4.9 μm for Li, 8.8 μm for Na, and 16.4 μm for K. Therefore, the thickness after first-cycle charge of the three different AFAMBs also varies at the same areal capacity (Fig. 1e) [37–39]. Although the overall cell-level thickness change also depends on stack pressure and other factors, the general trend remains clear: AFLMBs usually undergo smaller volume changes than the other two systems, whereas AFPMBs are expected to experience the most severe volume expansion. Such volume fluctuations can significantly affect interfacial stability, leading to repeated rupture and reconstruction of the SEI, loss of electrical contact, and accelerated formation of electrically isolated dead alkali metal. Therefore, although Li-, Na-, and K-based AFAMBs share the same configuration, they should not be regarded as directly equivalent systems because important differences remain in their deposition behavior, volume change, and interfacial stability.

## Important Performance Metrics for Practical AFAMBs

Systematic evaluation of AFAMBs is essential to understand their failure mechanisms and support their commercialization. Therefore, practical assessment should go beyond cycle number alone and explicitly consider metrics that reflect true alkali metal inventory retention, usable cathode capacity, and cell-level energy-density benefit under realistic conditions. In this regard, Coulombic efficiency (CE), active inventory retention, depth of discharge (DOD), and cell-level energy density are among the most informative performance descriptors for AFAMBs.

### Coulombic Efficiency and Alkali Metal Inventory Retention

CE is one of the most fundamental performance metrics for AFAMBs because these systems operate without an excess alkali metal reservoir on the anode side [40, 41]. As a result, every irreversible plating/stripping event directly consumes the limited inventory initially stored in the cathode and accelerates capacity decay. This sensitivity is much more severe than in excess metal configurations, where part of the irreversible loss can be buffered by surplus metal on the anode side [42, 43]. Accordingly, CE in AFAMBs should be evaluated not only as a measure of interfacial reversibility, but also as a full-cell metric closely related to alkali metal inventory retention. Initial CE, average CE, capacity retention, and inventory retention descriptors capture different levels of irreversibility in AFAMBs. The initial CE mainly reflects irreversible alkali loss during the first plating/stripping process, including initial interphase formation and other early side reactions, and therefore determines how much cyclable inventory remains after the first cycle. In contrast, the average CE over extended cycling reflects the reversibility of repeated plating/stripping and governs the rate at which the remaining active alkali metal inventory is consumed during subsequent cycles. Capacity retention represents a macroscopic full-cell outcome that integrates active alkali loss, cathode degradation, electrolyte consumption, impedance growth, and interfacial contact loss. Therefore, although capacity retention is closely related to CE and inventory retention, it should not be treated as a direct substitute for these metrics.

**Fig. 2:**
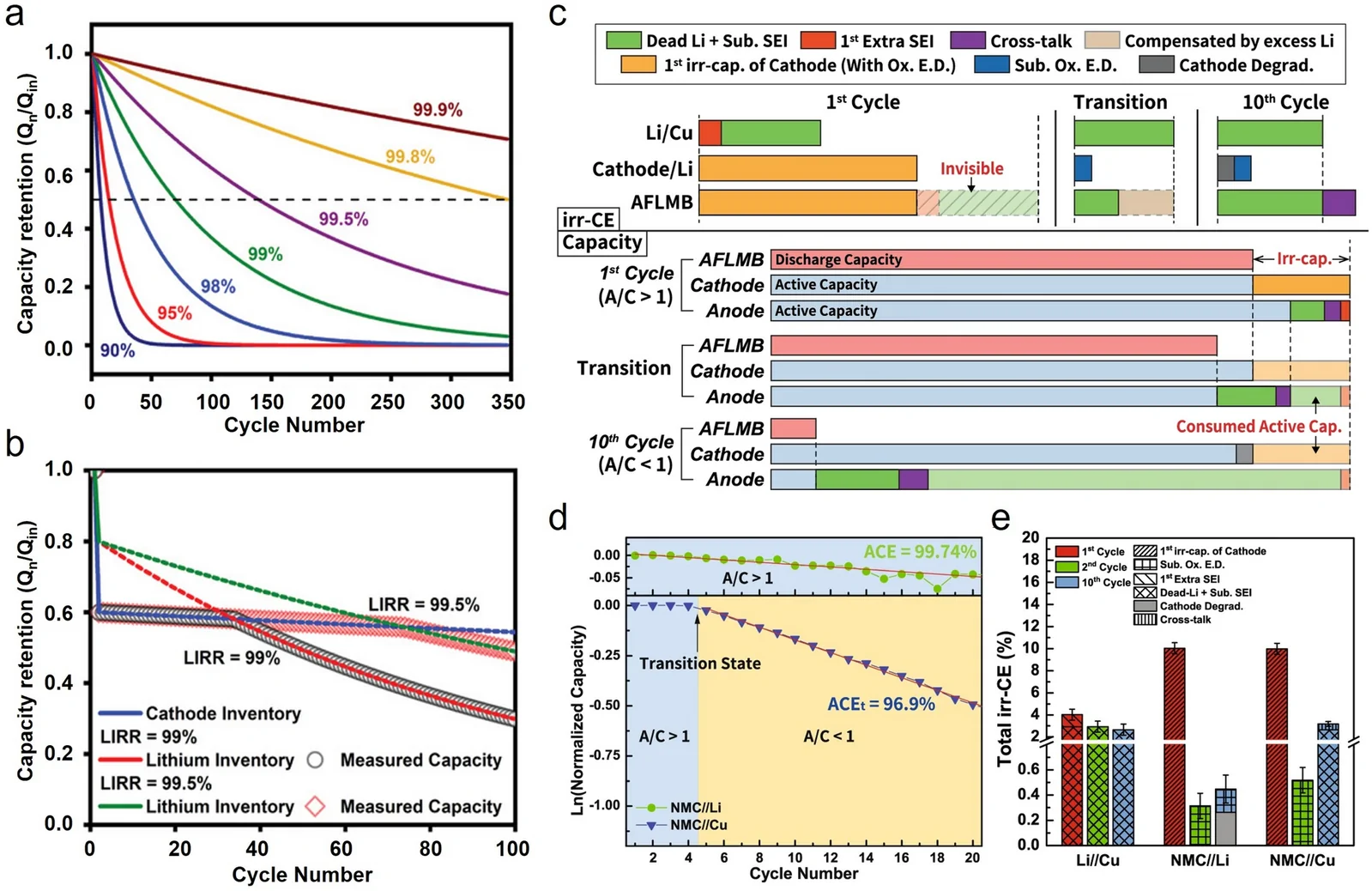
Interpreting AFAMB performance through CE and alkali metal inventory retention. **a** Idealized cycling performance of anode-free cells with different average CE values. **b** Simulated cycling behavior of anode-free full cells with an initial cathode inventory loss of 40% and a lithium inventory loss of 20% in the first cycle. Reproduced with permission [44]. Copyright 2020, Wiley-VCH. **c** Integrated protocol for deconvoluting the origins of irreversible CE in anode-free cells. **d** Capacity retention of Li||NCM111 and Cu||NCM111. **e** Comparison of the contributions of different irreversible factors to total capacity loss in different cells. Reproduced with permission [45]. Copyright 2021, Springer Nature

Manthiram and co-workers illustrated the idealized cycling behavior of anode-free cells with different average CE values, highlighting that even a small increase in CE could lead to a marked extension of cycle life (Fig. 2a) [44]. However, this analysis was based on a simplified assumption of constant CE during cycling. In practical cells, CE is not fixed, but continuously evolves with interphase growth, dead alkali metal accumulation, and changes in deposition/stripping morphology. Therefore, interpreting AFAMB performance solely based on a few early-cycle CE values can provide an incomplete picture of long-term cell behavior. To better describe alkali metal inventory loss during cycling, they further proposed the lithium inventory retention rate (LIRR), defined as LIRR = 100 *(*Q*_*n*_/*Q*_in_)^1/*n*^, where *Q*_*n*_ is the discharge capacity at cycle * n* and *Q*_in_ is the initial discharge capacity. This parameter provides a direct description of the average rate of cyclable Li retention during repeated cycling. As shown in Fig. 2b, for anode-free cells with an initial cathode inventory loss of 40% and a lithium inventory loss of 20%, the early-stage capacity is primarily limited by the cathode inventory. As cycling proceeds, the progressive depletion of Li inventory becomes increasingly dominant. Under these conditions, a cell with an LIRR of 99% exhibits rapid Li inventory depletion within 40 cycles, whereas a cell with a higher LIRR shows a much later onset of severe Li deficiency, at around 70 cycles. These results illustrate that seemingly small differences in average inventory retention efficiency can produce substantial divergence in long-term cycling stability. LIRR provides a useful descriptor for the average retention of cyclable alkali metal inventory, but its quantitative comparison requires consistent definitions of the initial capacity, discharge capacity basis, and cycle number. Therefore, CE and LIRR should be compared only together with the corresponding testing conditions and capacity retention profiles.

Beyond average CE or LIRR, the concept of irreversible CE was also introduced to deconvolute the major sources of capacity loss in AFLMBs [45]. In this framework, multiple contributors were considered, including dead Li formation, SEI growth, and irreversible cathode capacity loss (Fig. 2c). As shown in Fig. 2d, e, when the anode-to-cathode capacity ratio (A/C) is larger than 1, capacity decay is mainly limited by cathode-side loss. Once the cell enters the transition regime with A/C < 1, the alkali metal inventory on the anode side becomes insufficient, and rapid capacity fading is dominated by dead Li accumulation and subsequent SEI reformation. Such analysis is valuable because it links macroscopic full-cell fading to specific internal loss pathways and helps clarify which degradation mode should be prioritized in cell design.

These analyses were developed mainly in AFLMBs, as lithium systems are currently the most mature within the anode-free family, but the underlying principles are broadly applicable across AFAMBs. The first-cycle CE determines how much cyclable inventory remains after initial interphase formation and irreversible side reactions, while the average CE over extended cycling governs how rapidly the remaining inventory is depleted thereafter. For LIBs, it is widely recognized that the average CE must approach 99.95% to support long cycle life under commercially relevant conditions. In anode-free cells, the requirement for high CE is also very stringent. Accordingly, meaningful evaluation of AFAMBs should jointly consider the initial CE, the average CE over extended cycling, and the corresponding capacity retention profile, since a seemingly high early CE may still be accompanied by rapid exhaustion of the limited cyclable alkali metal inventory.

### Depth of Discharge

A lower DOD has been widely reported to improve battery performance, primarily because it helps preserve the cathode structure and mitigates structural degradation associated with deep cycling [46, 47]. Notably, this effect is even more pronounced in anode-free batteries. Under a lower DOD, part of the alkali metal inventory remains on the anode side after discharge, which can serve as an additional inventory reservoir for subsequent cycles. As a result, the cycle life of anode-free batteries can be substantially prolonged under shallow-discharge conditions. Furthermore, recent studies have shown that lowering the DOD can suppress dead Li formation and thereby slow alkali metal inventory depletion.

**Fig. 3:**
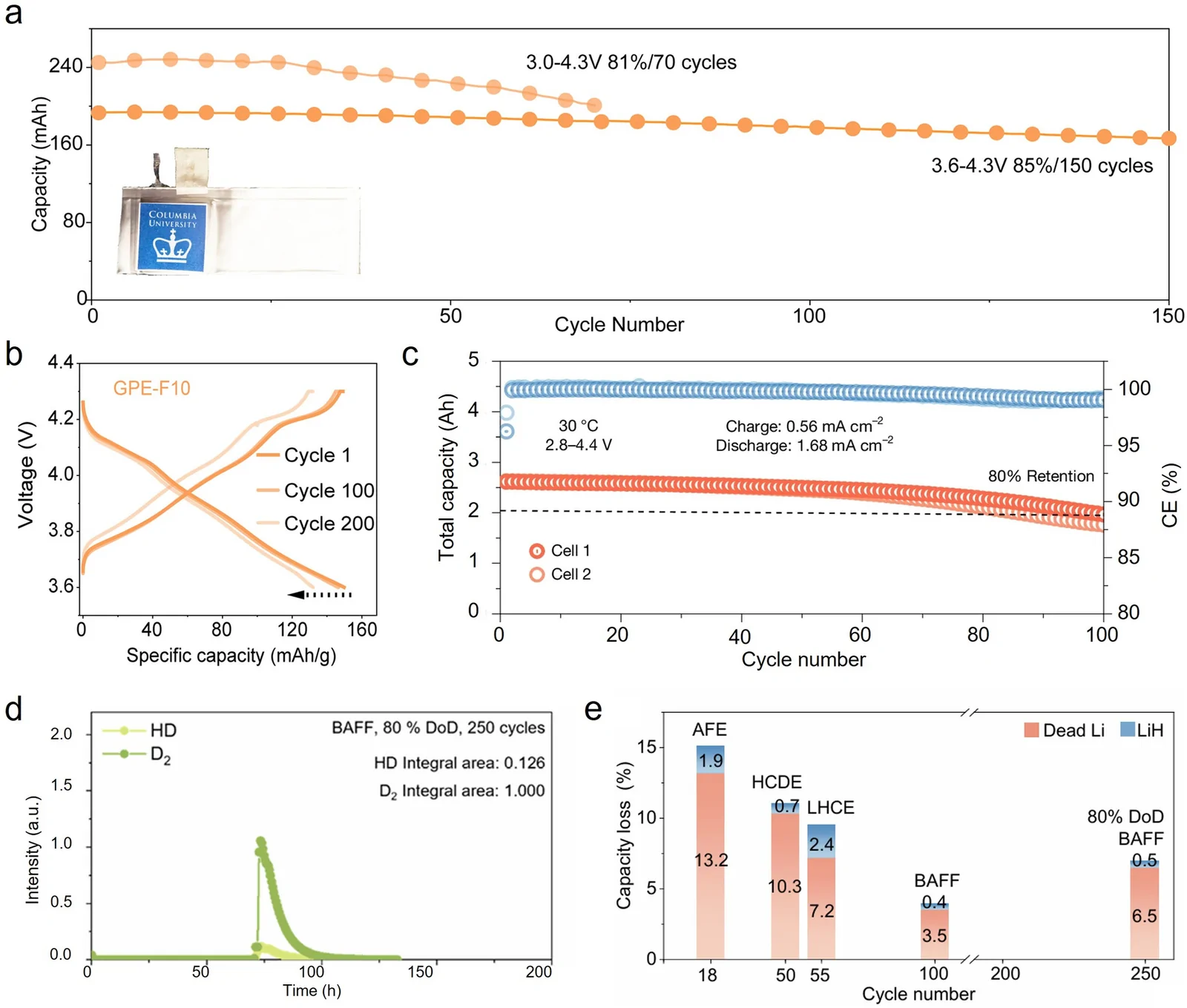
Influence of DOD on cycling performance in anode-free batteries. **a** Cycling performance of Cu||NCA pouch cells cycled in the 3.0–4.3 V and 3.6–4.3 V windows. **b** Voltage profiles of the Cu||NCA cell cycled in the 3.6–4.3 V window. Reproduced with permission [49]. Copyright 2026, Elsevier. **c** Cycling performance of Cu||NCM811 pouch cells using BAFF in the 2.8–4.4 V window. **d** MST-based quantitative analysis of inactive Li metal and LiH at 80% DOD. **e** Contributions of dead Li and LiH to capacity loss as determined by MST. Reproduced with permission [30]. Copyright 2026, Springer Nature

The effect of DOD is clearly illustrated in recent anode-free pouch-cell studies [30, 48, 49]. Dahn and co-workers reported Cu||NCM523 pouch cells that retained 80% of their capacity after 90 cycles when cycled within a 3.6–4.5 V window [48]. In another study, Yang and co-workers showed that a 240 mAh Cu||NCA anode-free pouch cell retained about 81.2% of its capacity after 70 cycles in the 3.0–4.3 V window, whereas narrowing the voltage window to 3.6–4.3 V extended the cycle life to 150 cycles (Fig. 3a) [49]. Figure 3b shows the voltage profile of the anode-free cell with a cutoff voltage of 3.6 V, where no abrupt terminal voltage drop is observed, indicating that the cell is not fully discharged. Similarly, Wang and co-workers assembled a 2.7 Ah anode-free pouch cell that retained 80% of its capacity after 100 cycles under full-depth operation in the 2.8–4.4 V window (Fig. 3c) [30]. When the discharge cutoff voltage was increased from 2.8 to 3.6 V, corresponding to 80% DOD, the cell capacity decreased to 2.16 Ah, but the cycle life was markedly prolonged to 250 cycles at 80% retention, representing one of the best results reported to date.

Wang et al. further quantified the amount of inactive Li using mass spectrometry-D_2_O titration (MST), as shown in Fig. 3d [30]. In their battery with 1.6 M LiDFOB/NDFA electrolyte (BAFF), dead Li and LiH accounted for 3.5% and 0.4% of capacity loss after 100 cycles (Fig. 3e). When they lowered the DOD to 80%, the amount of dead Li decreased to 0.001 mAh cm^−2^ per cycle, confirming that reduced DOD effectively suppressed irreversible Li loss. Therefore, in this alkali metal-limited system, lowering the DOD not only alleviates cathode-side structural degradation, but also slows alkali metal inventory depletion and the associated formation of dead metal and parasitic interphase.

However, the benefit of a lower DOD comes at the expense of energy output and partially offsets the energy-density advantage of AFAMBs because the active inventory is not fully utilized in each cycle. Therefore, long cycle life achieved under shallow-discharge conditions should not be directly compared with full-depth cycling results without considering the corresponding usable capacity and practical energy-density penalty. Since DOD is strongly affected by cathode chemistry, voltage window, cathode loading, and cell format, it should be explicitly reported when comparing AFAMB performance across different studies. For meaningful comparisons of AFAMB performance, DOD should be reported explicitly alongside areal capacity, electrolyte amount, stack pressure, voltage window, and the cell-level energy-density basis. Long cycle life achieved through partial capacity utilization should also be interpreted in light of the corresponding practical energy-density penalty.

Although the above examples are mainly based on AFLMBs, the underlying inventory retention logic of DOD is broadly relevant to AFAMBs because Li-, Na-, and K-based anode-free cells all operate with limited alkali metal inventory. Lowering DOD can preserve part of the plated alkali metal on the anode side after discharge and reduce irreversible inventory loss in subsequent cycles. However, this evaluation framework should not be directly transferred among AFLMBs, AFSMBs, and AFPMBs without considering chemistry-specific differences. The voltage window used to define DOD depends strongly on cathode chemistry and electrolyte stability in each system. Therefore, DOD-dependent cycling performance should be interpreted together with the specific alkali metal chemistry, cathode chemistry, voltage window, usable capacity, and energy-density penalty, rather than being directly compared across Li-, Na-, and K-based AFAMBs.

### Cell-Level Energy Density

Cell-level energy density remains a central motivation for AFAMB development. In practice, the energy-density advantage of AFAMBs can be realized when high-loading cathodes, lean electrolyte conditions, and minimal inactive interfacial modifications are achieved simultaneously. However, many reported improvements in cycling stability are achieved under relatively relaxed conditions, such as low cathode loading and excess electrolyte. Recent analyses have also noted that many reported anode-free cells are still evaluated in coin cells or small pouch cells with relatively low areal loading, making it difficult to directly assess their practical relevance. Among the three AFAMB systems, the highest publicly reported energy densities reached 508 Wh kg^−1^ and 1668 Wh L^−1^ in a 2.7 Ah AFLMB pouch cell [30]. For AFSMBs, a 4.3 V-class pouch cell delivered an energy density of 441.7 Wh kg^−1^, calculated based on the active materials of both electrodes [50]. AFPMBs remain at an earlier stage, with representative anode-free full cells achieving up to 362 Wh kg^−1^ based on the combined mass of the cathode and current collector [20]. Notably, many reported energy-density values for AFAMBs are calculated based only on active materials, rather than on the total cell weight. Therefore, these reported values should be interpreted in the context of their specific calculation basis and cell format, rather than being directly compared across studies.

## Recent Current Collector Designs to Improve Cycling Performance of AFAMBs

Since the current collector serves as the substrate for the in situ formed working anode in AFAMBs, recent progress has increasingly relied on chemistry-specific current collector engineering to regulate alkali metal nucleation, homogenize ion flux, accommodate volume change, and stabilize the interphase. The main degradation pathways in AFAMBs are closely associated with interfacial instability during metal plating and stripping. Heterogeneous alkali metal nucleation and growth on bare collectors can generate porous or filamentary deposits, increasing the risk of electrically isolated dead metal and incomplete stripping. Repeated plating and stripping further induce large volume fluctuations, which can lead to SEI cracking, continuous interphase reconstruction, electrolyte consumption, and progressive alkali metal inventory loss. In addition, weak adhesion between the plated metal and the current collector can cause void formation, delamination, and loss of electronic contact, thereby accelerating dead metal accumulation. The migration of cathode-derived species or oxidized electrolyte fragments to the anode side can further increase interfacial impedance and aggravate full-cell capacity decay. Current collector design can address these degradation pathways by homogenizing local current distribution, lowering the nucleation barrier, promoting dense and uniform deposition, accommodating volume fluctuation, stabilizing the SEI, and maintaining intimate contact with the plated alkali metal. Table 1 summarizes the main functions, key design criteria, and potential limitations of the different design strategies. In the following subsections, we briefly summarize representative current collector designs for AFLMBs, AFSMBs, and AFPMBs.

**Table 1:** Comparison of representative current collector modification strategies for AFAMBs

Strategy	Main functions	Key design criteria	Potential limitations
Artificial SEI/Protective coating	Stabilize interphase chemistry, suppress parasitic electrolyte reduction, and protect the plated alkali metal during cycling.	High alkali-ion conductivity, electronic insulation or regulation, chemical stability against electrolyte and alkali metal, mechanical compliance, strong adhesion, ultrathin thickness, and scalable fabrication.	Excessive thickness can increase polarization and inactive mass; poor adhesion or mechanical mismatch may cause cracking, delamination, or repeated SEI reconstruction.
Metal-philic nucleation seeds	Lower alkali metal nucleation barriers and guide spatially uniform initial deposition.	Uniformly distributed lithiophilic/sodiophilic/potassiophilic sites, appropriate binding strength, good electronic contact, low loading, and stable surface chemistry.	Uneven seeds may induce localized deposition, increase inactive mass, or become additional side-reaction sites.
Alloy-forming layers	Improve metal affinity, interfacial adhesion, and stripping reversibility through alloying/dealloying reactions.	Reversible alloying behavior, limited volume change, stable phase evolution, strong collector–metal contact, ultralow loading, and minimal irreversible alkali consumption.	Thick or unstable alloy layers may consume alkali metal inventory, increase interfacial resistance, or shift the cell away from a genuine anode-free configuration.
Morphology engineering	Reduce local current density, provide internal space for metal deposition, and accommodate volume fluctuation.	Low volume change, high electronic conductivity, accessible pore network, optimized pore size/tortuosity, mechanical robustness, and efficient ion/electron transport pathways.	Bulky structures, dead volume, or high surface area may reduce cell-level energy density, increase electrolyte consumption, and complicate manufacturing.

### Current Collector Design Strategies for AFLMBs

Among AFAMBs, AFLMBs are the most intensively studied and already provide a relatively mature framework for current collector engineering [51–56]. Compared with sodium and potassium, lithium offers the lowest redox potential and metal density, which translates into the highest achievable cell-level energy density when paired with high-loading layered oxide cathodes. In addition, the entire LIB manufacturing infrastructure, including Cu foil handling and electrolyte formulation, can be directly leveraged for anode-free architectures. These advantages make Li the natural starting point and the reference case for understanding how current collectors should be designed for AFAMBs. At the same time, Li has a very high reactivity and a strong tendency to form filamentary or porous deposits when it nucleates on a bare Cu foil under lean electrolyte and high areal capacity. Any isolated or electrically disconnected Li immediately becomes dead Li and leads to capacity loss in an anode-free configuration. Therefore, in AFLMBs, the current collector should enable highly reversible Li deposition on bare Cu under lean electrolyte and high areal capacity, while minimizing dead Li formation and interfacial instability from the very first cycle.

**Fig. 4:**
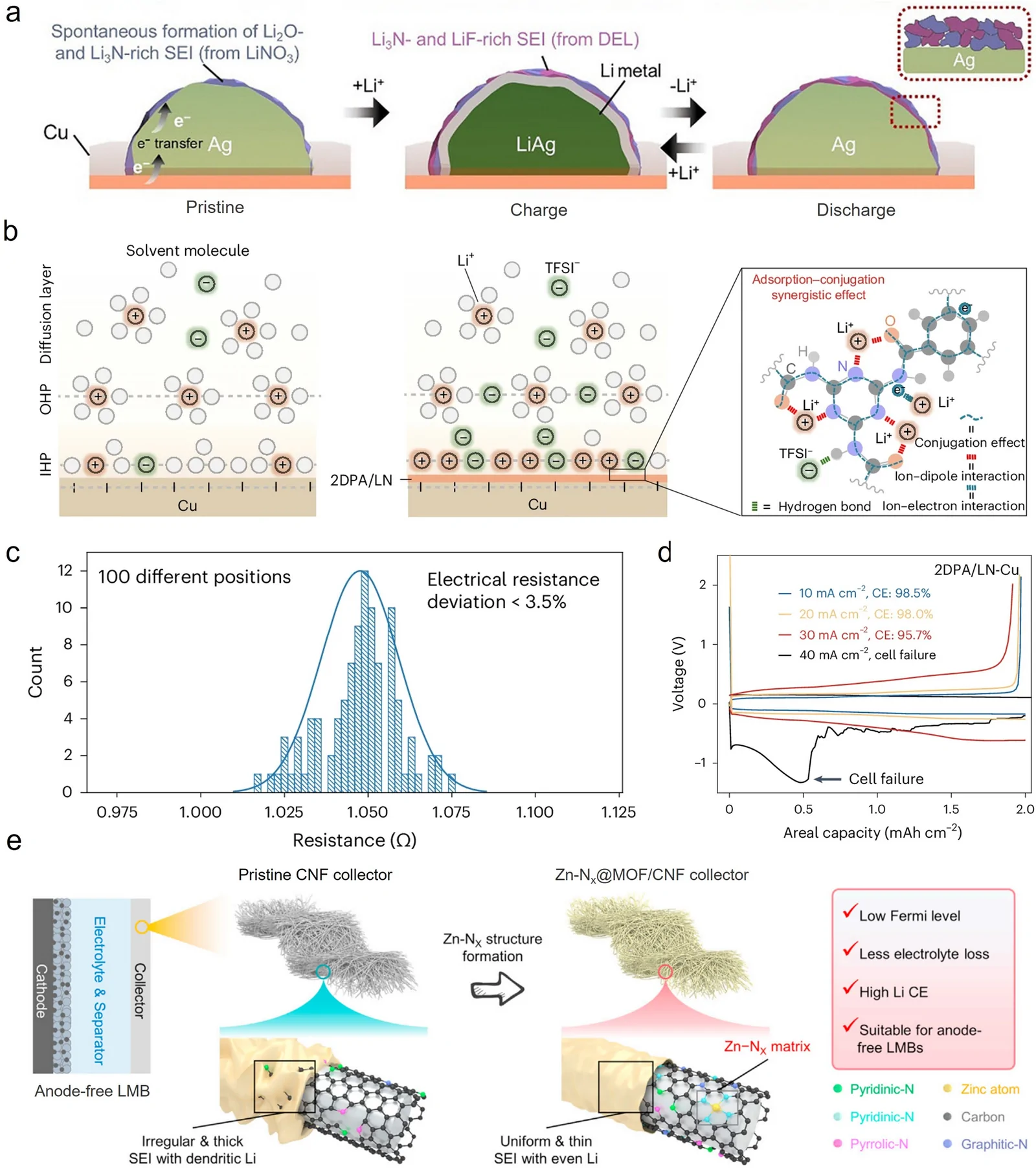
Interfacial and structural current collector designs for AFLMBs. **a** Schematics of the reversible host in the pristine, charge, and discharge states. Reproduced with permission [58]. Copyright 2025, Wiley-VCH. **b** Schematic view of interfacial double layers for bare Cu and 2DPA/LN-Cu. The IHP, OHP (outer Helmholtz plane), and diffusion layer are demonstrated. **c** Electrical resistance measured at 100 different positions on 2DPA/LN-Cu. **d** Voltage profiles of 2DPA/LN-Cu half-cells at different current densities. Reproduced with permission [60]. Copyright 2025, Springer Nature. **e** Schematic of the advantages of Zn-N-CNF current collector. Reproduced with permission [62]. Copyright 2024, Elsevier

Cui and co-workers systematically compared Li nucleation on various metal substrates [57]. Substrates such as Sn, Au, Ag, and Zn exhibited little or no nucleation overpotential because they formed Li-rich alloys or displayed partial mutual solubility at the interface, indicating high lithiophilicity. Dai et al. employed a Si-PAN coating on Cu foil to enhance anode-free battery performance [53]. The resulting current collector formed a stable Li–Si interface and showed good affinity for Li^+^ due to the N-rich PAN component. Park and co-workers prepared a reversible host (RH) layer composed of Ag nanoparticles to accommodate the volume change during cycling by Li–Ag alloying and dealloying (Fig. 4a) [58]. In combination with their carbonate-rich electrolyte, the RH layer further promoted the formation of a stable Li_2_O- and Li_3_N-rich SEI through synergistic coupling. These alloy-forming designs should be interpreted beyond simple nucleation overpotential reduction. In anode-free cells, alloying layers can regulate the spatial distribution of initial nuclei, strengthen the adhesion between deposited Li and the current collector, and improve stripping reversibility by maintaining interfacial contact during repeated alloying/dealloying. However, their function depends strongly on loading, reversibility, and structural stability. Alloy layers may improve initial Li wetting or interphase formation, but they can irreversibly consume the limited Li inventory if the alloy reaction is not sufficiently reversible. Reversible alloy hosts, such as Ag-based layers, can buffer volume change and improve Li utilization, but excessive loading or unstable phase evolution may introduce additional polarization and inactive mass. Ultrathin alloy coatings or patterned alloy seeds are therefore more attractive for preserving the genuine anode-free configuration. Multicomponent alloy layers and bilayer or gradient designs may further balance metal affinity, electronic conductivity, mechanical compliance, and interphase stability, but their added processing complexity, interfacial resistance, and manufacturability should be carefully evaluated under lean-electrolyte and high-areal-capacity conditions.

Polymer coatings and artificial SEI have also attracted considerable attention in this field. Most polymer interphases are designed to be electrochemically inert toward Li, thereby avoiding unnecessary consumption of active Li. Tamwattana et al. employed a LiF@PVDF layer as an artificial SEI to protect active Li and promote reversible Li plating/stripping [59]. Sun and co-workers prepared a two-dimensional polyamide with lithiated Nafion (2DPA/LN) as an interphase layer (Fig. 4b) [60]. The adsorption-conjugation effect of the 2DPA/LN modulated the inner Helmholtz plane (IHP), which in turn facilitated Li^+^ transport and homogenized its interfacial distribution. Importantly, the introduction of the 2DPA/LN layer did not compromise the electrical conductivity of the current collector (Fig. 4c), and the resulting half-cell could sustain current densities as high as 30 mA cm^−2^ (Fig. 4d). Benefiting from these interfacial advantages, they demonstrated an 8 Ah pouch cell delivering an energy density of 471 Wh kg^−1^ and a power density of 622 W kg^−1^. Kim et al. revealed that a pre-SEI can spontaneously form on Cu current collectors during the resting stage before Li plating [61]. This pre-SEI is governed by electrolyte composition and originates from the preferential reduction of electrolyte-derived anion–cation aggregates at the Cu surface. This pre-SEI regulates subsequent Li nucleation morphology, suppresses dendritic growth, and improves cycling stability, highlighting that current collector/electrolyte interfacial chemistry can be decisive even before the working Li metal anode is formed. Taken together, these examples indicate that artificial SEI layers or protective coatings should actively regulate interfacial transport and reactions, rather than simply covering the current collector surface. An effective coating should allow sufficient Li^+^ transport while limiting electron leakage into the electrolyte, thereby suppressing continuous electrolyte reduction. It should also remain chemically stable against both the electrolyte and freshly deposited Li, while maintaining mechanical compliance and strong adhesion to tolerate repeated volume change. For practical anode-free cells, these functions should be achieved with ultrathin, low mass, and scalable coatings, so that the added interfacial protection does not offset the intrinsic energy-density advantage of the anode-free configuration.

Beyond the Cu-based current collectors, carbon-based current collectors are widely explored because their 3D architecture provides more space to accommodate Li growth. Kwon et al. synthesized multivacancy-deficient carbon paper, thereby lowering the Fermi level of the current collector [51]. The lower Fermi level of the current collector suppressed electron transfer from the collector to the lowest unoccupied molecular orbital of the electrolyte, thereby reducing electrolyte decomposition. Another representative approach was based on carbon nanofiber (Fig. 4e) [62]. A low-Fermi-level collector (Zn-N-CNF) was obtained by electrospinning a mixture of PAN and Bio-MOF-100. The high affinity of the Zn-N-CNF for anions promoted the formation of a thin and ionically conductive SEI, and the Zn-containing lithiophilic sites reduced Li nucleation overpotential and homogenized the interfacial current distribution. The modified collectors enabled dense Li deposition, while the corresponding symmetric cells operated for more than 6000 h at 5 mA cm^−2^ and 5 mAh cm^−2^.

Overall, research on Li-based anode-free cells suggests that anode-free configurations represent a promising strategy for addressing the rapidly increasing demand for high-energy-density storage. Lithium’s combination of high theoretical energy density and SEI chemistry has enabled a diverse framework of collector designs. Recent research on AFLMBs focuses on improving interphase stability and promoting uniform Li nucleation/deposition and stable stripping.

### Emerging Current Collector Designs for AFSMBs

Compared with AFLMBs, AFSMBs have lower material costs and broader resource availability, making them particularly attractive for large-scale energy storage [18, 50, 63–65]. Many Na cathodes use Fe/Mn-rich layered oxides or Prussian blue analogs. Na shows a much weaker tendency to alloy with Al than Li within the practical electrochemical window, allowing Al-based collectors to behave as inert current collectors in AFSMBs. This suppresses parasitic alloying reactions while reducing the cost and mass of current collectors, making Al-based collectors a key advantage for constructing low-cost AFSMB systems [66–69]. By contrast, AFLMBs typically rely on heavier and more expensive Cu foils because Li readily forms Li–Al alloys within the practical anode potential window, which can irreversibly consume cyclable Li and compromise the structural integrity of the current collector. However, this cost and collector-compatibility advantage does not directly translate into facile Na metal reversibility. Compared with Li, Na has a larger ionic radius, different solvation/desolvation behavior, and generally slower interfacial kinetics, which make dense and reversible Na deposition more difficult under anode-free conditions. In addition, the larger plated Na thickness at the same areal capacity causes more pronounced volume fluctuation and imposes stronger mechanical stress on the SEI and collector–metal interface. Therefore, current collectors in AFSMBs should simultaneously lower Na nucleation barriers, promote fast Na^+^ transport, accommodate deposition-induced strain, and maintain stable interfacial contact during repeated plating/stripping [70–74]. These requirements explain why recent AFSMB collector designs increasingly emphasize Al-compatible porous hosts, sodiophilic interfacial sites, ion transport-regulating layers, and mechanically robust carbon-based frameworks.

**Fig. 5:**
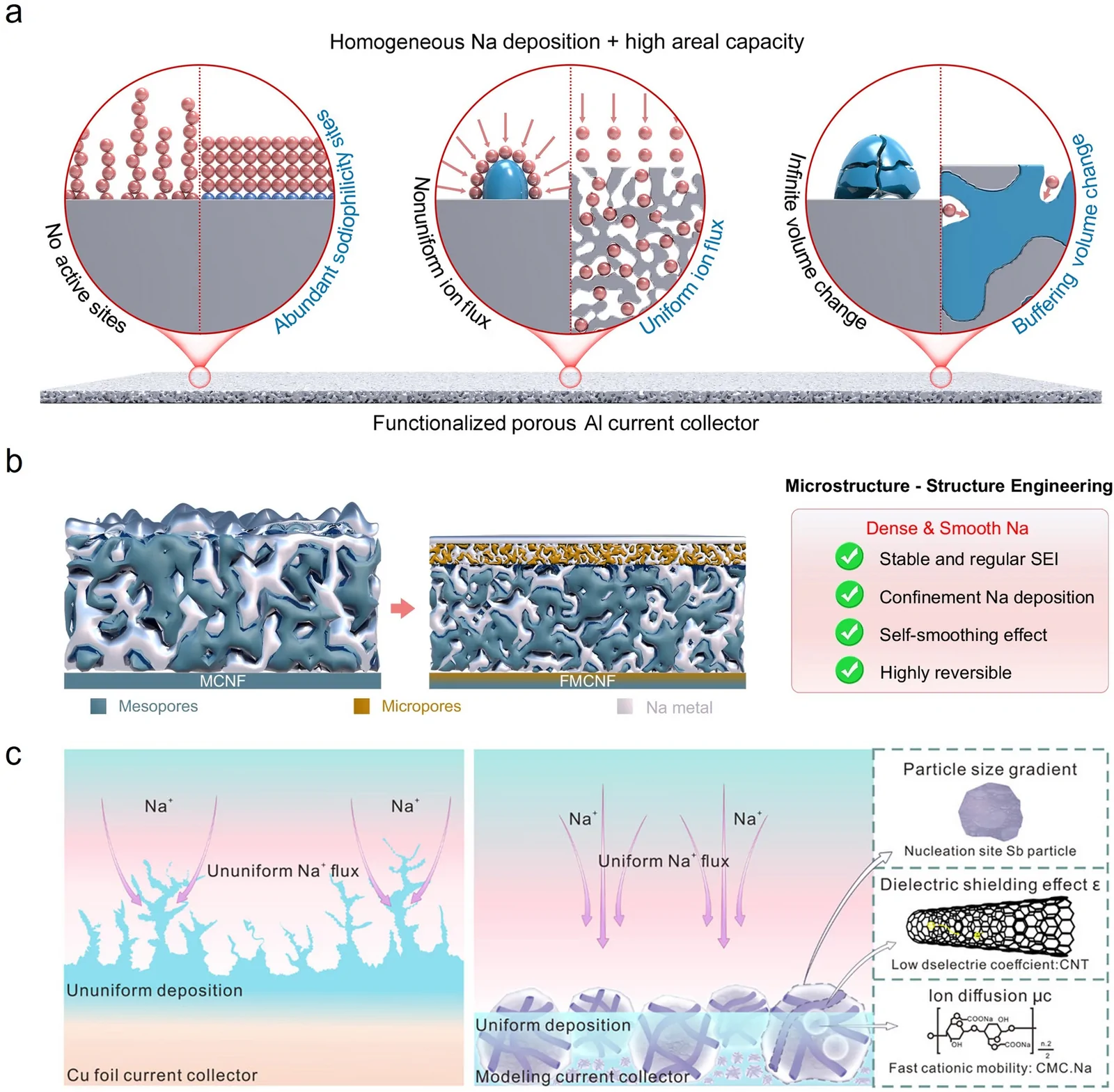
Al-compatible and 3D host designs for AFSMBs. **a** Main features of normal Al and porous Al current collectors. Reproduced with permission [75]. Copyright 2025, American Association for the Advancement of Science. **b** Microstructures and deposited Na morphologies of MCNF and FMCNF current collectors. The strengths of FMCNF are summarized in the table on the right. Reproduced with permission [76]. Copyright 2025, Springer Nature. **c** Schematic illustration of the Na growth process on Cu foil and the designed current collectors. Reproduced with permission [79]. Copyright 2025, Wiley-VCH

A representative example was the functionalized porous Al current collector reported by Lou and co-workers (Fig. 5a) [75]. Template-free electrodeposition followed by physical separation yielded an interconnected ligament–channel network with abundant sodiophilic sites, which strengthened Na adsorption, accelerated charge transfer, and lowered the nucleation overpotential. The porous scaffold reduced local current density and homogenized Na^+^ flux, suppressing tip amplification and mitigating stress from Na volume changes, thereby enabling highly reversible Na plating and stripping. Under anode-free conditions, this porous Al host enabled pouch cells with about 420 Wh kg^−1^ and about 84.9% capacity retention after 100 cycles, highlighting the potential of lightweight Al hosts for high-energy AFSMBs.

Carbon-based 3D current collectors also enable concurrent control of Na nucleation, SEI chemistry, and mechanical robustness in AFSMBs. Zhu et al. designed fluorine-doped, micropore-coated mesoporous carbon nanofibers (FMCNFs) (Fig. 5b), where F and Zn–N_x_ moieties form Lewis-acidic and sodiophilic centers that suppressed electrolyte decomposition and guided Na to uniformly distributed nucleation sites [76]. The obtained FMCNFs further yielded a thin, compliant SEI and an ultralow nucleation overpotential. As a result, FMCNFs sustained over 5000 plating/stripping cycles with an average CE of 99.93% at 5 mA cm^−2^. Another study developed N, P-doped macroporous carbon fibers decorated with CoP nanoparticles (CoP@N/P-CMFs) as foldable current collectors [77]. The macroporous fiber network buffered plating strain and, together with sodiophilic CoP and N, P-doped sites, enabled dendrite-free Na deposition with CE above 99.97% at 10 mA cm^−2^ and 10 mAh cm^−2^.

Besides bulk host design, interfacial functionalization of conventional metal foils has emerged as an efficient route to achieve high-rate AFSMBs. Hu et al. in situ grew a Prussian blue analog (PBA) layer on Cu foils to form a fast Na ion diffusion interlayer (PBA@Cu) [78]. The open PBA framework provided low diffusion barriers to Na^+^ diffusion and accelerated ion transport across the interface. It homogenized Na⁺ flux at the collector surface and regulated interfacial anion chemistry, thereby promoting the formation of an inorganic-rich SEI with enhanced ionic conductivity and mechanical robustness. With this architecture, anode-free full cells maintained stable cycling at 5 C for 300 cycles, illustrating that fast ion transport interlayers can push AFSMB operation into the ultrafast regime without sacrificing stability. Zhang et al. proposed a quantitative design framework for AFSMB current collectors based on a descriptor k [79]. This composite parameter combines the dielectric coefficient of the interfacial region, the ion mobility within the host and the change in local Na concentration. It provides a dimensionless descriptor for current collector performance, and smaller k indicates more favorable conditions for uniform Na deposition. Guided by this descriptor, they optimized a carbon nanotube host modified with sodium carboxymethyl cellulose (Fig. 5c). The resulting collector sustained Na plating and stripping for more than 24 months at 0.25 mAh cm^−2^ and 0.5 mA cm^−2^. The good agreement between the measured performance and the k-based predictions highlights the potential of this approach for future current collector design. More broadly, this descriptor-based study suggests that effective current collector design should not rely on a single property alone. Instead, uniform alkali metal deposition requires the coordinated regulation of interfacial polarization, ion transport, and local concentration gradients.

Overall, recent progress in AFSMBs reveals a design logic that is partially shared with AFLMBs but also distinctly sodium-specific. While homogeneous nucleation, strain buffering, and interphase stabilization remain central goals, AFSMBs place greater emphasis on low-cost Al-compatible collectors, robust Na deposition under larger volume fluctuations, and accelerated Na^+^ transport across fragile interfaces. These considerations collectively define a practical framework for current collector engineering in sodium-based anode-free batteries.

### Early-Stage Current Collector Development for AFPMBs

Benefiting from the high natural abundance of K and its relatively low redox potential, AFPMBs are also emerging as promising alternatives to lithium systems for stationary and grid-scale storage. However, compared with AFLMBs and AFSMBs, AFPMBs remain the least developed and arguably the most challenging. In addition, the industrial demand and supply chain for metallic K remain limited, which further slows the development and practical translation of K-based batteries. The mechanistic understanding of K deposition under anode-free conditions is still limited [19, 80]. Relative to Li and Na, K has a larger ionic radius, lower volumetric capacity, and stronger interfacial reactivity. These features make current collector design for AFPMBs fundamentally different from that for Li- and Na-based systems. At the same areal capacity, K plating requires a thicker metal layer, which imposes larger interfacial fluctuations and stronger mechanical stress on the collector–metal interface [81, 82]. Meanwhile, the higher reactivity of K increases the tendency for parasitic electrolyte decomposition and unstable SEI formation. Therefore, AFPMB current collectors should not only provide potassiophilic sites to lower the K nucleation barrier, but also regulate interfacial electron transfer, suppress excessive electrolyte reduction, accommodate large K deposition volume, and maintain stable electrical contact during repeated plating/stripping. Because K already suffers from a lower volumetric capacity, these functions should be achieved without introducing excessive inactive mass or dead volume.

**Fig. 6:**
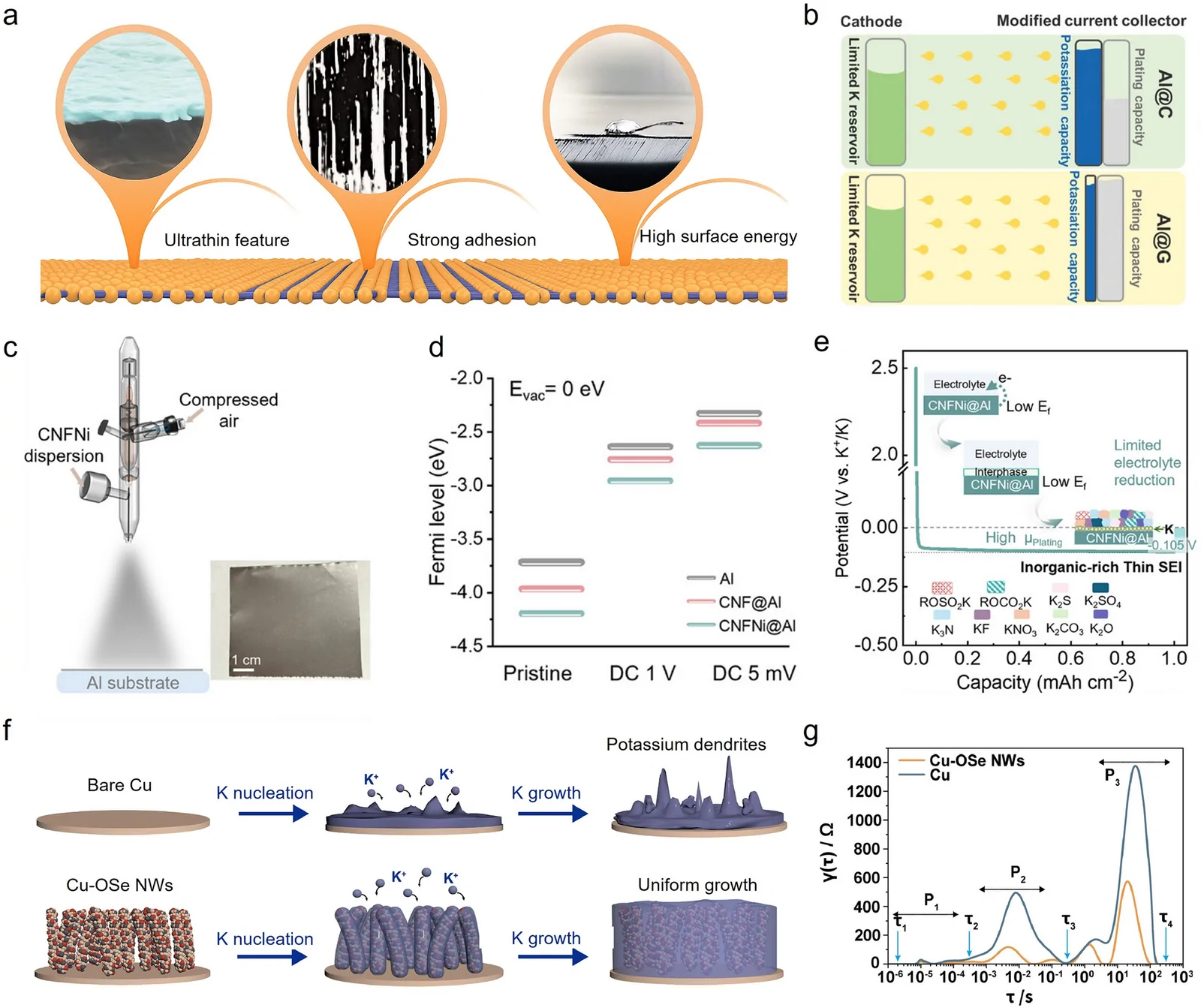
Surface-engineered and potassiophilic host designs for AFPMBs. **a** Features of Al@G current collectors. **b** Schematic illustration of active K loss on different current collectors. Reproduced with permission [83]. Copyright 2022, Wiley-VCH. **c** Spray painting of CNFNi on Al to produce CNFNi@Al. **d** Fermi levels of different current collectors based on work function. **e** SEI formation process on CNFNi@Al upon first-cycle K deposition in a three-electrode system. Reproduced with permission [84]. Copyright 2025, Wiley-VCH. **f** Schematic illustration of K nucleation and deposition on bare Cu and Cu-OSe NWs. **g** Distribution of relaxation times analysis of anode-free full cells based on bare Cu and Cu-OSe NWs. Reproduced with permission [85]. Copyright 2024, Elsevier

Zhao et al. demonstrated that directly growing a graphene skin on commercial Al current collectors (Al@G) mitigated the potassiophobic nature of Al and enabled anode-free operation [83]. The conformal graphene layer improved electronic homogeneity and potassiophilicity while preserving mechanical robustness (Fig. 6a). Consequently, Al@G exhibited suppressed dendrite growth and improved reversibility (Fig. 6b), supporting current densities up to 4.0 mA cm^−2^ and areal capacities up to 4.0 mAh cm^−2^, as well as stable cycling for about 1000 h at 0.5 mA cm^−2^ and about 750 h under periodically fluctuating currents (0.1–2.0 mA cm^−2^). More recently, surface chemistry tuning has emerged as another effective approach to regulate electrolyte decomposition and interphase properties [84]. Zhang and co-workers coated Al with a thin Ni-decorated carbon nanofiber layer to decrease the collector Fermi level and suppress parasitic solvent reduction (Fig. 6c, d). The resulting SEI was inorganic-rich (Fig. 6e), mechanically resilient toward K volume changes, and ionically conductive, while Ni sites served as potassiophilic nucleation centers that homogenized K deposition. This result showed that current collector surface engineering could effectively complement electrolyte design, enabling stable operation of a 4.4 V-class AFPMB in a conventional-concentration electrolyte.

Potassiophilic 3D hosts represent another effective route to control K nucleation and growth under anode-free conditions. Cui et al. designed partially selenized Cu-OSe nanowires (Cu-OSe NWs) as a potassiophilic scaffold for AFPMBs (Fig. 6f) [85]. The partially selenized Cu-OSe sites lowered the K nucleation barrier, while the low K^+^ migration barrier along the nanowire surface facilitated rapid surface diffusion. Consistently, distribution of relaxation times analysis revealed improved interfacial kinetics in the Cu-OSe NW-based anode-free full cells compared with those using bare Cu (Fig. 6g). As a result, K||K symmetric cells using Cu-OSe NWs exhibited highly reversible K plating/stripping for 950 h at 1 mA cm^−2^ and 1 mAh cm^−2^. In anode-free full cells, this host extended the cycle life to about 200 cycles, highlighting that appropriately engineered mixed anion frameworks can serve as both nucleation seeds and ion transport highways for K deposition. Li et al. proposed a customized combination of a diluted high-concentration electrolyte and a mesoporous carbon nanofiber host (MCNF) to simultaneously address interphase chemistry and deposition morphology [20]. The diluted high-concentration electrolyte with fast kinetics and high stability promoted the formation of an inorganic-rich and durable SEI, while MCNF with narrowly distributed mesopores offered a reduced external surface area yet sufficient internal free volume for K accommodation. This co-design delivered an average CE of 99.3% and an initial CE of 95.9% at 3 mA cm^−2^ and 3 mAh cm^−2^ and enabled anode-free MCNF||Prussian blue full cells with 100 reversible cycles and a high energy density of about 362 Wh kg^−1^.

Emerging strategies combine surface chemistry and electronic tuning to suppress parasitic reduction with structured hosts that homogenize local current distribution and accommodate K growth. Nevertheless, AFPMB studies remain relatively limited, and Ah-class demonstrations are still lacking. Moving forward, establishing broadly applicable design descriptors and validating performance under practical conditions, including high areal capacity, lean electrolyte, and Ah-class full cells, will be essential for translating these advances into durable and high-energy AFPMBs.

Table 2 summarizes representative recent studies on AFAMBs enabled by current collector engineering across Li, Na, and K systems. Although cell configurations and testing conditions vary across reports, several broad trends are clear. Most reported cells still exhibit cycling lifetimes below 500 cycles, while high-rate and Ah-class demonstrations remain relatively scarce. Moreover, some of the reported long cycle performances are obtained under relatively mild testing conditions, such as low cathode loading or excess electrolyte, and therefore may not fully translate to practical cell configurations. These comparisons highlight that AFAMBs, despite their rapid progress, still require substantial improvements in energy density, durability, and practical validation before broad commercialization can be realized. To further synthesize the design implications from these studies, Table 3 summarizes the broadly applicable current collector design principles and the chemistry-dependent implementation priorities for Li-, Na-, and K-based anode-free cells. In addition to the intrinsic properties of current collectors, collector−electrolyte compatibility should be considered as a key factor in AFAMBs. Before a stable working alkali metal anode is formed, the electrolyte is in direct contact with the bare or modified current collector surface. Therefore, early electrolyte reduction, surface passivation, and corrosion reactions can influence the initial interphase chemistry and subsequent metal nucleation. Electrolyte solvation structures and desolvation barriers also affect the local ion supply near the current collector, while anion-derived decomposition products can determine whether the resulting interphase is mechanically robust, ionically conductive, and chemically stable. These effects are especially important in anode-free cells, where limited alkali metal inventory leaves little tolerance for continuous electrolyte decomposition and irreversible metal loss.

**Table 2:** The cycling performance comparison of AFAMBs employing current collector engineering

	Current collector	Cathode	Voltage (V)	Cathode loading (mg cm^−2^)	Energy density (Wh kg^−1^)	Rate/ Current density	Cycle	Capacity retention (%)	References
AFLMBs	Cu@Si-PAN	LiNi_0.5_Mn_1.5_O_4_	3.0–4.85	1.2		50 mA g^−1^	120	80	[53]
	2DPA/LN-Cu	NCM811	3.0–4.3	5.6	471	0.3/0.5 C	20	93.3	[60]
	Cu/Ag/LiNO_3_	NCM811	2.5–4.2	4.6	346.7	0.2 C	80	73.9	[58]
	Zn-N-CNF	LFP	2.4–4.0	2.07		0.5 C	120	91	[62]
	CP-SrI_2_	NCM532	2.5–4.2			0.5 C	200	82	[14]
	D-CP	NCM811	2.7–4.3	4.2		0.48 C	50	90	[51]
	Ag-Cu	NCM811	3.0–4.3	5	450	0.2/0.5 C	100	85	[54]
	TEG-Cu	LFP	2.0–3.8	3	527	0.3/0.5 C	50	80	[88]
	Cu-HPBF	NCM811	3.0–4.3	4.1		1 C	67	80	[55]
	HEA-C	NCM811	3.0–4.3	3.4		1 C	160	56.67	[56]
AFSMBs	Porous Al	NVOPF	2.0–4.3	0.67	420.4	65 mA g^−1^	100	84.9	[75]
	CoP@N/P-CMFs	NVP	2.0–3.0	1.58		1 C	100	67.1	[77]
	FMCNF	NVOPF	2.6–3.8	2.24	383	0.3 C	200	90	[76]
	N-Sb@M-Sb@CNT@C-Na	NVP	2.3–3.6	3.97	387.6	0.33 C	175	93.9	[79]
	UGB-Cu	NVP	3.0–3.6	0.78	357.2	1 C	250	75.71	[70]
	D-ZnO	NVOPF	2.0–4.3	0.62	441.7	65 mA g^−1^	105	86	[50]
	SnNCNF	NVP	2.6–3.8	0.8		2 C	80	89	[71]
	FCTF	NVP	2.5–3.8	0.84		240 mA g^−1^	400	56	[72]
	Al~HC	NFPP	1.5–3.5	2	203	0.2 C	180	95.2	[73]
	Cu_3_P	NVP	2.5–3.5			0.5 C	170	51.7	[74]
AFPMBs	Al@G	K-Fe_2_S	0.8–3.0			100 mA g^−1^	30		[83]
	CNFNi@Al	MnHCF	2.5–4.4			100 mA g^−1^	50	89	[84]
	Cu-OSe	K_0.5_MnO_2_	1.5–3.9			100 mA g^−1^	200	63.5	[85]
	MCNF	Prussian blue	2.0–4.4		362	20 mA g^−1^	100	86	[20]

**Table 3:** Universal and chemistry-specific design considerations for current collector engineering in AFAMBs

Category	Summary
Universal principles for AFAMBs	Uniform nucleation, dense deposition, stable interphase, reversible stripping, low inactive mass, and scalable manufacturing are broadly required across Li-, Na-, and K-based anode-free cells.
AFLMB-specific emphasis	AFLMBs provide the most extensively studied platform for current collector engineering, with particular emphasis on translating Li-affinitive surface modification, interphase regulation, and dead Li suppression into lean-electrolyte, high-areal-capacity, and pouch-cell conditions without introducing excessive inactive mass.
AFSMB-specific emphasis	AFSMBs require current collectors and interfacial chemistries that support uniform Na nucleation, fast Na^+^ transport, and stable collector–Na contact. These requirements are especially important because Na deposition can involve larger thickness variation, more fragile interphases, and more pronounced strain/contact loss during repeated plating and stripping.
AFPMB-specific emphasis	AFPMBs demand potassiophilic yet low-mass interfaces that can suppress electrolyte reduction, tolerate large K volume fluctuation, and maintain mechanical/electrochemical stability. Strict inactive-mass control is particularly important because of the lower volumetric capacity and stronger interfacial reactivity of K metal.

## Key Parameters for Current Collector Design in AFAMBs

**Fig. 7:**
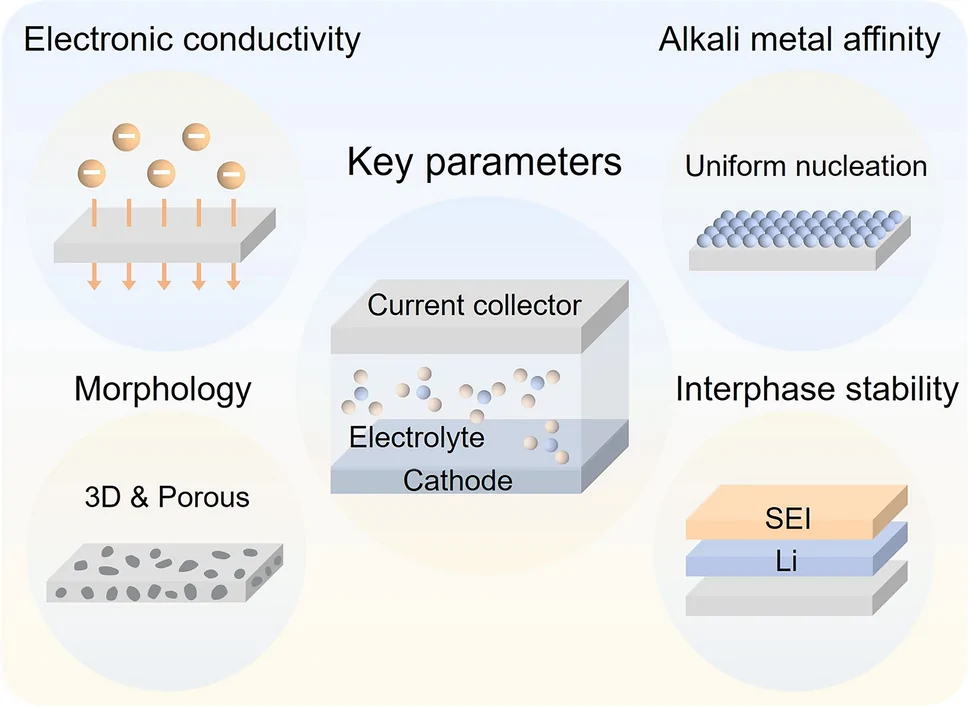
Schematic illustration of the four essential parameters governing current collector design in AFAMBs

Li-, Na-, and K-based AFAMBs differ in electrochemical behavior, interfacial chemistry, and practical design priorities. Nevertheless, current collector designs across these systems follow several shared principles [86–88]. These principles can be summarized into four interrelated parameters: electronic conductivity, alkali metal affinity, collector morphology, and interphase stability (Fig. 7), with their implementation adapted to the specific alkali metal chemistry.

### Electronic Conductivity

Sufficient electronic conductivity is a primary requirement for current collectors in AFAMBs, as it dictates ohmic losses and the maximum achievable rate capability at practical areal capacities [62, 89, 90]. Low-resistance pathways are essential to homogenize the in-plane potential distribution, thereby avoiding local current hotspots that trigger non-uniform alkali metal deposition. At the same time, the introduction of functional coatings or porous scaffolds should not compromise the overall conductivity of the collector; otherwise, the apparent energy and power advantages of AFAMBs may be offset by increased polarization and reduced round-trip efficiency. Therefore, electronic conductivity should be explicitly considered in current collector design, particularly when polymeric or metal compound-based coatings/architectures are introduced, because these materials may exhibit lower conductivity than pure metallic collectors and can increase internal resistance.

In modified current collectors, factors such as coating thickness, transport continuity, contact resistance, and structural integrity during cycling can all influence the effective electron pathway. Appropriate design should maintain rapid and continuous charge transport while enabling additional functions such as affinity regulation or interphase control. Therefore, conductivity optimization should be considered together with architectural and interfacial design so that functional modification does not come at the expense of excessive resistance, inactive mass, or limited rate performance.

### Alkali Metal Affinity

The affinity between the current collector surface and alkali metal critically controls the nucleation overpotential and the spatial distribution of nascent nuclei [57, 75, 90, 91]. Consequently, engineering alkali metal affinity is a central and unavoidable theme in AFAMB design. Surfaces with appropriate lithiophilicity, sodiophilicity, or potassiophilicity can lower the nucleation barrier, promote dense and uniform nucleation, and suppress filamentary growth. Conversely, overly inert surfaces lead to high nucleation overpotentials and sparse nucleation sites, which amplify local current densities and favor mossy or dendritic growth. Engineering the surface chemistry and electronic structure of current collectors to achieve controlled alkali metal affinity is therefore essential for enabling uniform nucleation in AFAMBs.

Such affinity is not an intrinsic constant of the collector surface but is determined by its surface chemistry and atomic/electronic structure. Specifically, alkali metal affinity is mainly governed by the surface composition, defect environment, exposed crystallographic facets, and other surface characteristics. In addition, the interfacial structure can further regulate charge transfer, electrolyte reduction, and the chemistry of the nascent interphase, thereby modifying the apparent alkali metal affinity during early deposition. Therefore, effective affinity engineering should not simply aim to maximize alkali metal binding, but rather to balance adsorption, surface diffusion, and interphase formation to realize uniform nucleation, dense lateral growth, and reversible plating/stripping in AFAMBs.

### Current Collector Morphology

The morphology of the current collector, particularly the adoption of 3D porous architectures, strongly influences local current distribution and the accommodation of plated alkali metal [92–94]. 3D scaffolds provide a larger surface area and internal free volume, which help decrease the local current density and buffer the large volumetric changes associated with metal plating and stripping. However, excessive thickness or dead volume in the collector region can introduce parasitic mass and reduce the cell-level energy density. Therefore, 3D architectures should be carefully designed to balance mechanical and kinetic benefits with stringent areal-mass constraints. The impact of current collector morphology further depends on structural parameters such as pore size, pore connectivity, thickness, and tortuosity, which together determine the accessibility of the internal framework to electrons and alkali ions. Appropriate structural design can promote uniform deposition and efficient accommodation of plated metal, while minimizing isolated deposits, incomplete stripping, and excess interphase formation. Accordingly, morphological engineering should focus on lightweight and structurally efficient architectures that provide sufficient deposition space without introducing excessive inactive mass.

In foil-based anode-free cells, planar surface engineering should be treated as a distinct design variable rather than simply as a simplified form of 3D host design. Surface roughness, crystallographic texture, micro/nano-patterning, and patterned metal-philic sites can all regulate the spatial distribution of local electric fields and nucleation sites [70, 95]. For example, controlled roughness or patterned surface features can distribute local current density and guide more homogeneous alkali metal deposition, while crystallographic texture and patterned lithiophilic, sodiophilic, or potassiophilic sites can lower nucleation barriers at predefined locations. Such planar engineering strategies are particularly attractive for practical AFAMBs because they can improve nucleation uniformity and deposition morphology while largely preserving the low mass, thin geometry, and manufacturing compatibility of conventional foil current collectors.

### Interphase Stability

The stability of the SEI is a decisive factor for the CE and cycle life of AFAMBs [96–98]. During repeated plating and stripping, large volume changes in the alkali metal impose substantial mechanical stress on the interphase, making it prone to cracking, void formation and delamination. These degradations can promote dead alkali metal formation and dendritic growth, ultimately leading to cell failure. Meanwhile, instabilities in the SEI consume active alkali metal and electrolyte and lead to progressive inventory loss and interfacial degradation. SEI evolution in AFAMBs should be viewed as a dynamic process that includes initial nucleation-coupled SEI formation, repeated rupture/reconstruction during plating and stripping, and long-term chemical and mechanical stabilization. The current collector participates in this process through its surface chemistry, electronic structure, alkali metal affinity, morphology, and adhesion with the plated metal. During the plating stage, a collector with appropriate alkali metal affinity can lower the nucleation barrier and homogenize metal deposition, thereby reducing localized electrolyte decomposition and promoting a more uniform SEI. During cycling, conductive and mechanically stable collector frameworks can help maintain intimate contact with the plated alkali metal, reduce void formation, and suppress repeated SEI fracture. Electronically regulated surfaces can reduce excessive electron leakage into the electrolyte and favor the formation of inorganic-rich SEI components with improved ionic conductivity and mechanical robustness.

The stability of the current collector–alkali metal interphase is also important. A weak interphase between the current collector and the deposited alkali metal can lead to the loss of electronic contact between the deposit and the substrate, or even complete detachment. Interphase stability further depends on both the chemical nature and the spatial uniformity of the interface. An interphase that is chemically unstable, mechanically fragile, or unevenly distributed can induce heterogeneous ion flux, localized stress accumulation, and repeated rupture/reconstruction during cycling. Accordingly, effective interphase engineering should not only aim to form inorganic-rich and mechanically robust SEI species, but also to maintain intimate and uniform contact between the plated metal and the current collector. In this regard, surface chemistry regulation, artificial interphase construction, and interfacial structural design can all play important roles in suppressing side reactions and preserving long-term interfacial stability.

**Fig. 8:**
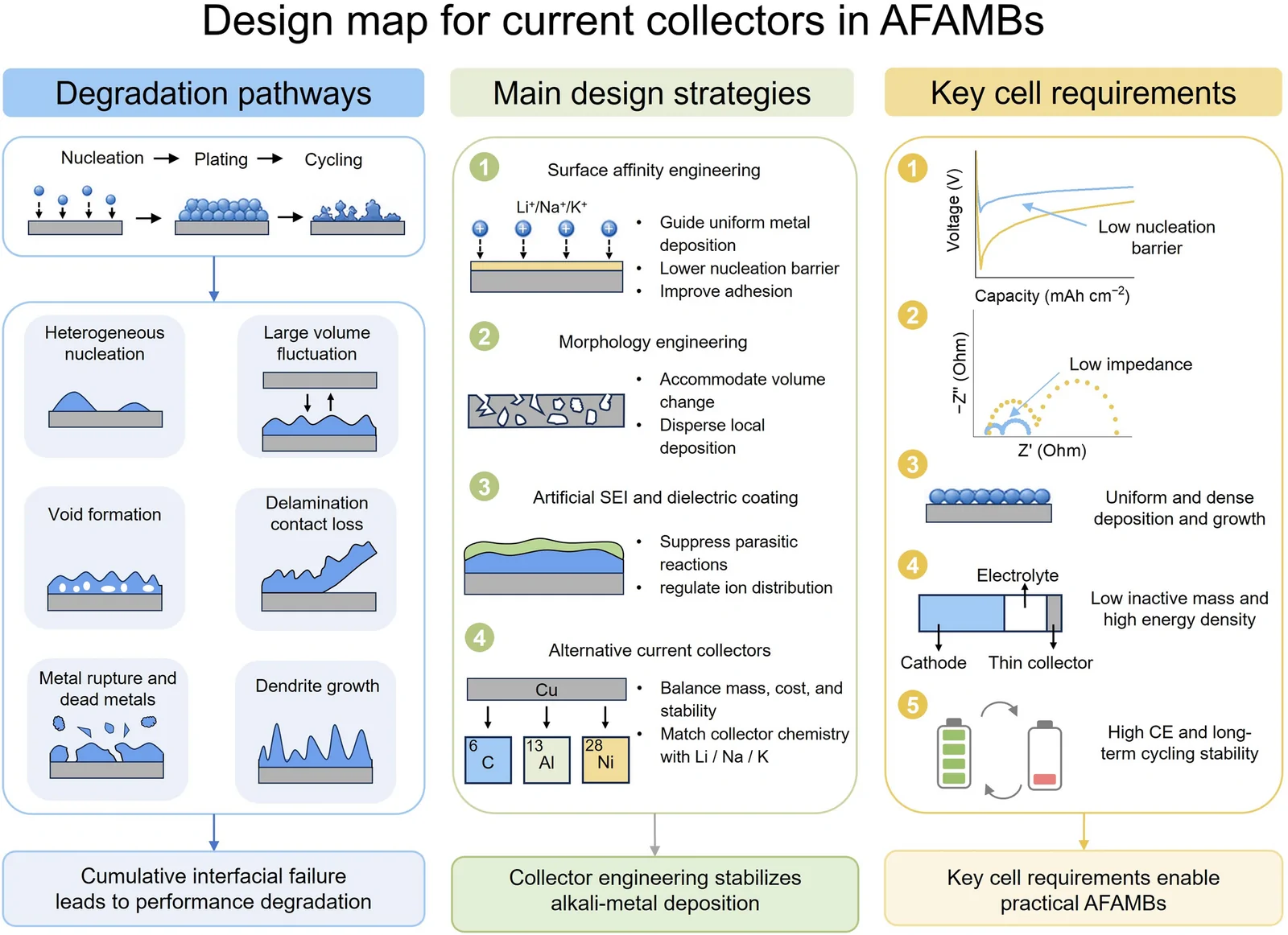
Design map for current collectors in AFAMBs

In summary, these four parameters are all critical to the cycling stability of AFAMBs. Furthermore, they are closely correlated rather than independent of one another. Electronic conductivity determines the uniformity of charge transport and local current distribution, thereby influencing alkali metal nucleation and growth. Alkali metal affinity regulates the nucleation barrier and the spatial distribution of initial metal nuclei, but its practical effect is further affected by the morphology and interfacial chemistry of the collector. Collector morphology can lower local current density and accommodate the volume change of plated alkali metal, whereas excessive thickness, tortuosity, or dead volume may compromise electron/ion transport and reduce cell-level energy density. Interphase stability is also coupled with these parameters, because non-uniform current distribution, sparse nucleation, and mechanically unstable deposition can promote repeated SEI rupture, parasitic reactions, and loss of electrical contact. Rational current collector design should thus aim to coordinate electronic transport, nucleation behavior, structural accommodation, and interfacial stability in an integrated manner. These interrelated considerations also provide a useful basis for interpreting recent current collector designs in Li-, Na-, and K-based anode-free batteries. Figure 8 illustrates the current collector design map in AFAMBs. During cycling, unstable current collector interfaces can induce non-uniform deposition, contact loss, inactive metal formation, and dendritic growth, leading to cumulative interfacial failure and performance degradation. Surface affinity engineering, morphology engineering, artificial SEI construction, and alternative current collectors provide complementary routes to stabilize the metal/current collector interface. These strategies are designed to meet key cell requirements, including low nucleation barriers, low interfacial impedance, uniform and dense metal deposition, high CE, and fast and uniform ion transport, ultimately supporting long cycle life and high energy density. By linking interfacial failure pathways with corresponding collector engineering strategies and cell-level requirements, this map provides a practical framework for rational current collector design in AFAMBs.

## Conclusion and Perspectives

This review summarizes recent current collector engineering strategies for practical AFAMBs and clarifies the design logic across Li-, Na-, and K-based systems. Although these anode-free chemistries share common requirements for stable nucleation, reversible deposition/stripping, interphase stability, and low inactive mass, their implementation should be adapted to the distinct interfacial chemistry and deposition behavior of each alkali metal. Recent progress in AFLMBs has been substantial, yet most reported cells are still evaluated at rates of ≤ 1 C, particularly in pouch formats. These gaps underscore an urgent need for high-rate, Ah-class pouch cells with robust reliability. Additionally, studies on AFPMBs remain relatively limited, and no Ah-class AFPMB has been reported to date. Overall, AFAMBs still face a nontrivial gap to commercialization. At the same time, the field is expanding rapidly, with growing participation from both academia and industry, suggesting that practical deployment may be closer than previously anticipated [99].

**Fig. 9:**
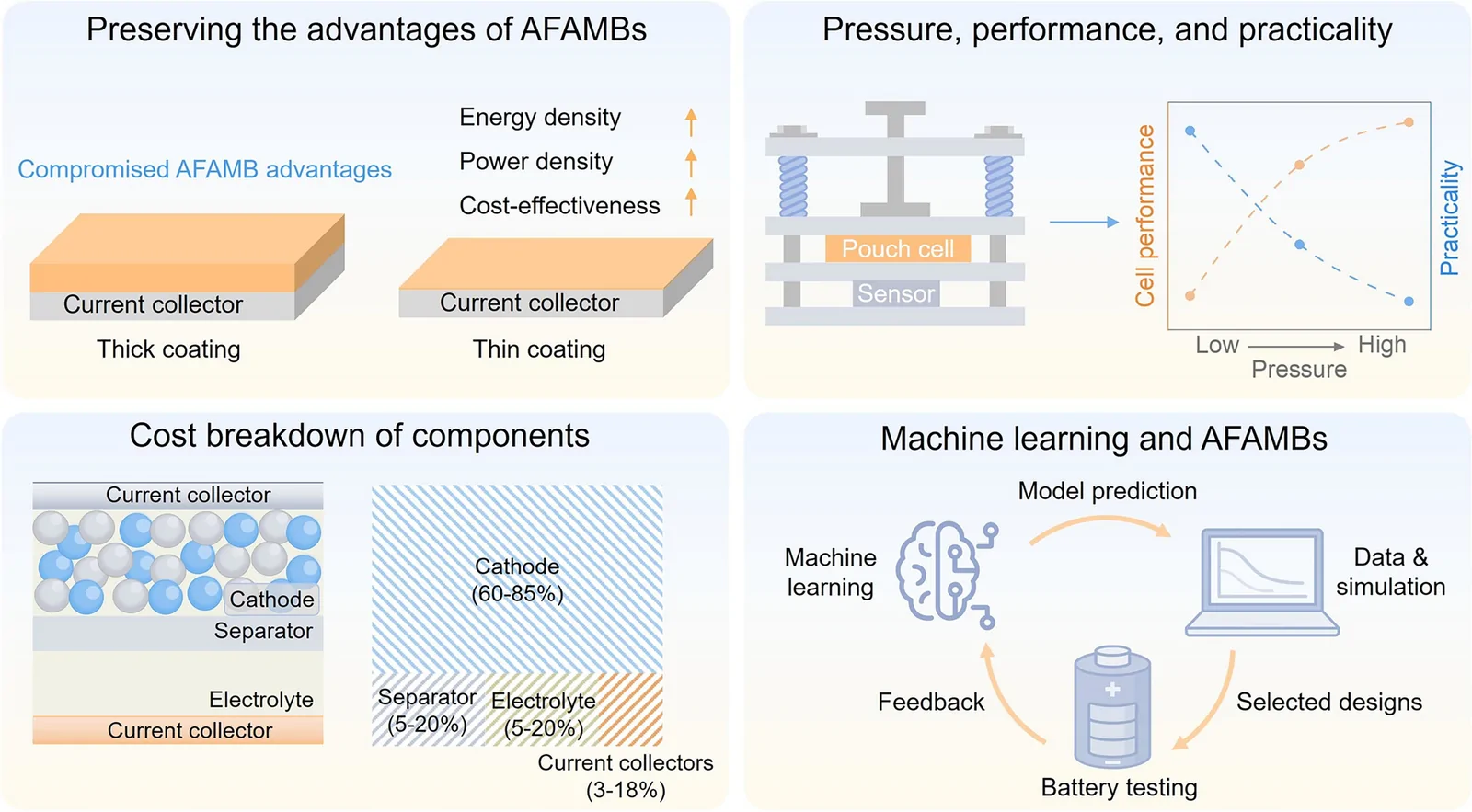
Key concepts and design principles of AFAMBs

Beyond the specific chemistries, it is also important to consider several broader perspectives on AFAMB design, particularly regarding genuine anode-free operation, electrochemical stability, and economic viability. Figure 9 summarizes these concepts and highlights four representative dimensions. The following subsections elaborate each of these aspects and outline their implications for practical AFAMB design.

### Preserving the Genuine Anode-Free Advantage through Limited Loading

A core design principle of AFAMBs is that the anode must remain essentially free of alkali metal at cell assembly to reduce cell mass and volume. Notably, some reported designs employed coatings with thicknesses comparable to or even greater than that of the current collector. Such approaches inevitably add substantial inactive mass/volume and shift the configuration toward a hybrid anode, thereby eroding the gravimetric and volumetric advantages that motivate the anode-free concept in the first place. Current collector engineering, protective coatings, and alloying strategies should employ nanometer-scale or otherwise ultrathin coatings, instead of introducing thick host layers that carry significant capacity. Additionally, the anode capacity should arise predominantly from the reversible plating and stripping of metallic Li, Na, or K, rather than from bulk intercalation or conversion reactions in metal compounds [100–102].

Therefore, coatings and alloying components are expected to act as functional interfacial modifiers instead of capacity contributors. They tune nucleation sites, lower interfacial overpotential, homogenize current distribution, or regulate solid electrolyte interphase formation, but their contributions to cell capacity and total cell mass should remain negligible at the cell level. This principle is particularly important when using metal-rich or redox-active compounds, where a clear distinction must be drawn between alkali metal affinity functions and unintended insertion or conversion reactions involving the alkali metal. Maintaining minimal loadings helps preserve the fundamental identity of AFAMBs.

### Stack Pressure as a Critical Design Parameter

Stack pressure is another critical but often underappreciated design variable in AFAMBs. Moderate compressive pressure improves interfacial contact, reduces impedance, and can suppress dendritic or filamentary growth by mechanically constraining the alkali metal [103–106]. Recent studies on both anode-free lithium and sodium cells show that increasing stack pressure markedly improves CE and cycling stability by promoting dense deposits and minimizing void formation [48, 107–109]. However, many laboratory-scale demonstrations now operate at pressures around 1 MPa or even higher, which are difficult to maintain in large-format commercial modules and may lead to unrealistic performance projections.

From a design perspective, stack pressure should be optimized within a bounded operating window rather than simply increased to maximize cycle life. Ideally, AFAMBs need to function reliably within a practical pressure window (< 300 kPa) that is compatible with industrial module and pack design, mechanical tolerances, and safety requirements. This also calls for materials and interfaces that can sustain dense metal deposition and stable stripping at moderate pressures, as well as standardized reporting of stack pressure and contact configuration. Moreover, stack pressure should be optimized in concert with the mechanical properties of the current collector, separator, and cathode, because excessive compression can trigger particle fracture and separator deformation, thereby undermining cell reliability.

### Cost and Manufacturability Constraints in AFAMBs

Cost and manufacturability play an important role in differentiating AFAMBs designed for practical deployment from idealized laboratory cells. On the materials side, the cathode typically accounts for the largest fraction of cell mass and cost (60–85%), although the exact breakdown among cathode, separator, electrolyte, and current collectors varies with chemistry and cell format [110–114]. AFAMB architecture therefore needs to be evaluated at the full cell and pack levels rather than only from the perspective of the anode side. At the same time, anode-free configurations reduce negative-electrode active mass but increase relative sensitivity to cathode price, electrolyte volume and formation protocols, so improvements in anode-side utilization should be considered in conjunction with cathode selection and the overall bill of materials.

From a current collector perspective, it is desirable for AFAMBs to rely on low-cost substrates and scalable coating technologies. The use of aluminum or carbon-based collectors in AFAMBs, as well as the modification of conventional copper foils with ultrathin functional layers, offers a low-barrier route for practical translation because these strategies largely retain current collector formats and cell architectures. Nevertheless, current collector engineering often adds materials and process complexity, especially when it involves vacuum deposition, high-temperature treatments or multistep patterning. As a result, the performance gains delivered by a given collector design should be carefully weighed against the additional costs in raw materials, equipment, throughput and yield. Beyond material cost and processing complexity, compatibility with the well-established Li-ion manufacturing infrastructure should also be considered when evaluating current collector designs, as this remains the most mature platform for practical battery production. Ideal modification strategies should remain compatible with commercial foil handling, electrode coating or surface treatment, drying, calendering, electrolyte filling, formation, and pouch-cell assembly. Therefore, the practical value of a current collector design should be assessed not only by laboratory-scale electrochemical performance, but also by its compatibility with existing manufacturing lines and its ability to preserve the genuine anode-free energy-density advantage at scale.

### Data-Driven and Machine Learning-Guided Design for AFAMBs

Artificial intelligence (AI) can help accelerate the development of AFAMBs by predicting long-term outcomes from early-cycle data and by optimizing operating windows under practical constraints. In Li-ion systems, data-driven models have already shown that cycle life can be predicted from early-cycle voltage/time-series features, establishing a template for prognosis using only the first tens to hundreds of cycles [115, 116]. The paradigm can be transferred to AFAMBs to forecast CE, evolution of voltage hysteresis, and failure risk before costly long-term testing. In parallel, closed-loop optimization has been demonstrated to efficiently search charging or formation protocols with machine learning, suggesting a route to tune AFAMB formation, stack pressure, and electrolyte-to-capacity ratio within explicit safety and manufacturability bounds.

Crucially, AFAMB-specific work now shows that active learning can directly accelerate electrolyte discovery for anode-free cells, reducing trial and error by iteratively proposing and testing candidates based on prior results [117, 118]. This demonstrates the value of AI not only for prognostics and protocol optimization but also for chemistry design in the anode-free context. Feature sets that combine early voltage/current descriptors, impedance parameters, and interfacial chemistry markers are particularly suitable for AFAMBs. Coupling these features with Bayesian optimization or active learning loops can prioritize the most promising conditions for coin and pouch cell validation while respecting limited alkali metal inventories.

## References

[1] X. Yang, H. Zhang, Q. Liu, G. Jiang, The Li-ion battery industry and its challenges. Nat. Rev. Chem. **9**(8), 497–498 (2025). 10.1038/s41570-025-00742-2 40646258

[2] C. Jin, O. Sheng, G. Wei, H. Li, Q. Han et al., Inhibiting and rejuvenating dead lithium in battery materials. Nat. Rev. Chem. **9**(8), 553–568 (2025). 10.1038/s41570-025-00722-6 40456922

[3] H. Liu, W.-H. Lai, H.-L. Yang, Y.-F. Zhu, Y.-J. Lei et al., Efficient separators with fast Li-ion transfer and high polysulfide entrapment for superior lithium-sulfur batteries. Chem. Eng. J. **408**, 127348 (2021). 10.1016/j.cej.2020.127348

[4] Z. Zhang, W.-Q. Han, From liquid to solid-state lithium metal batteries: fundamental issues and recent developments. Nano-Micro Lett. **16**(1), 24 (2024). 10.1007/s40820-023-01234-y PMC10661211 37985522

[5] H. Liu, L. Zhao, P. Jiang, X. Sun, J. Hester et al., La(OH)_3_-based lithium ionic conductor for quasi-solid-state lithium metal batteries. Adv. Mater. **38**(8), e16709 (2026). 10.1002/adma.202516709 41318914

[6] H. Liu, W. Pei, W.-H. Lai, Z. Yan, H. Yang et al., Electrocatalyzing S cathodes *via* multisulfiphilic sites for superior room-temperature sodium–sulfur batteries. ACS Nano **14**(6), 7259–7268 (2020). 10.1021/acsnano.0c02488 32433868

[7] H. Yang, Z. Sun, Y. Zhao, Y. Zhang, Z. Sun et al., Flash joule heating-induced spinel-phase surface in Ni-rich layered oxide positive electrodes to stabilise lattice oxygen. Nat. Commun. **17**, 4008 (2026). 10.1038/s41467-026-70616-8 PMC13136373 41834008

[8] Z. Sun, J. Zhang, J. Zhang, C. Zhang, J. Han et al., Synergistic structural and defect engineering in MoS_2_ featuring ultra-expanded interlayers for fast-chargeable and long-durable sodium-ion batteries. Adv. Mater. **38**(7), e17606 (2026). 10.1002/adma.202517606 41267486

[9] L. Dong, S. Zhong, S. Zhang, B. Yuan, J. Liu et al., Toward practical anode-free lithium pouch batteries. Energy Environ. Sci. **16**(12), 5605–5632 (2023). 10.1039/d3ee01599h

[10] Y. Chen, C. Ye, N. Zhang, J. Liu, H. Li et al., Prospects for practical anode-free sodium batteries. Mater. Today **73**, 260–274 (2024). 10.1016/j.mattod.2024.01.002

[11] S. Zhou, Z. Sun, J. Zhang, H. Yang, Z. Sun et al., Single-atom engineering for synergistic nucleation and interfacial regulation enabling durable anode-free sodium metal batteries. Adv. Mater. **38**(7), e13154 (2026). 10.1002/adma.202513154 41229209

[12] H. Liu, W.-H. Lai, Y. Liang, X. Liang, Z.-C. Yan et al., Sustainable S cathodes with synergic electrocatalysis for room-temperature Na–S batteries. J. Mater. Chem. A **9**(1), 566–574 (2021). 10.1039/d0ta08748c

[13] H. Liu, W.-H. Lai, Q. Yang, Y. Lei, C. Wu et al., Understanding sulfur redox mechanisms in different electrolytes for room-temperature Na–S batteries. Nano-Micro Lett. **13**(1), 121 (2021). 10.1007/s40820-021-00648-w PMC8096878 34138346

[14] L. Dong, S. Zhong, B. Yuan, Y. Li, J. Liu et al., Reconstruction of solid electrolyte interphase with SrI_2_ reactivates dead Li for durable anode-free Li-metal batteries. Angew. Chem. Int. Ed. **62**(23), e202301073 (2023). 10.1002/anie.202301073 37011095

[15] G. Deysher, J.A.S. Oh, Y.-T. Chen, B. Sayahpour, S.-Y. Ham et al., Design principles for enabling an anode-free sodium all-solid-state battery. Nat. Energy **9**(9), 1161–1172 (2024). 10.1038/s41560-024-01569-9

[16] Y. Qiao, H. Yang, Z. Chang, H. Deng, X. Li et al., A high-energy-density and long-life initial-anode-free lithium battery enabled by a Li_2_O sacrificial agent. Nat. Energy **6**(6), 653–662 (2021). 10.1038/s41560-021-00839-0

[17] J. Huang, X. An, Z. Cheng, L. Li, S.-X. Dou et al., Dipole-dipole interaction-induced dense primitive solid-electrolyte interphase for high-power Ah-level anode-free sodium metal batteries. Nat. Commun. **16**, 8586 (2025). 10.1038/s41467-025-63593-x PMC12479860 41022750

[18] F. Tang, Y. Yang, C. Liu, S. Yang, S. Xu et al., Initially anode-free sodium metal battery enabled by strain-engineered single-crystal aluminum substrate with (100)-preferred orientation. Nat. Commun. **16**(1), 2280 (2025). 10.1038/s41467-025-57424-2 PMC11889270 40055318

[19] M. Tang, S. Dong, J. Wang, L. Cheng, Q. Zhu et al., Low-temperature anode-free potassium metal batteries. Nat. Commun. **14**, 6006 (2023). 10.1038/s41467-023-41778-6 PMC10522645 37752165

[20] S. Li, H. Zhu, C. Gu, F. Ma, W. Zhong et al., Customized electrolyte and host structures enabling high-energy-density anode-free potassium–metal batteries. ACS Energy Lett. **8**(8), 3467–3475 (2023). 10.1021/acsenergylett.3c01067

[21] J. Shi, D. Wang, Q. Liu, Z. Yu, J.-Q. Huang et al., Intermetallic layers with tuned Na nucleation and transport for anode-free sodium metal batteries. Nano Lett. **25**(5), 1800–1807 (2025). 10.1021/acs.nanolett.4c04282 PMC11803737 39870493

[22] J. Chen, J. Xiang, X. Chen, L. Yuan, Z. Li et al., Li_2_S-based anode-free full batteries with modified Cu current collector. Energy Storage Mater. **30**, 179–186 (2020). 10.1016/j.ensm.2020.05.009

[23] Y. Liu, X. Meng, Y. Shi, J. Qiu, Z. Wang, Long-life quasi-solid-state anode-free batteries enabled by Li compensation coupled interface engineering. Adv. Mater. **35**(42), 2305386 (2023). 10.1002/adma.202305386 37460207

[24] S.E. Sandoval, C.G. Haslam, B.S. Vishnugopi, D.W. Liao, J.S. Yoon et al., Electro-chemo-mechanics of anode-free solid-state batteries. Nat. Mater. **24**(5), 673–681 (2025). 10.1038/s41563-024-02055-z 39748055

[25] Z. Lu, H. Yang, Q.-H. Yang, P. He, H. Zhou, Building a beyond concentrated electrolyte for high-voltage anode-free rechargeable sodium batteries. Angew. Chem. Int. Ed. **134**(20), e202200410 (2022). 10.1002/ange.202200410 35226757

[26] Y. Xin, M. Zhu, H. Zhang, X. Wang, High-entropy materials: a new paradigm in the design of advanced batteries. Nano-Micro Lett. **18**(1), 1 (2026). 10.1007/s40820-025-01842-w PMC12267778 40670862

[27] H. Wu, S. Luo, H. Wang, L. Li, Y. Fang et al., A review of anode materials for dual-ion batteries. Nano-Micro Lett. **16**(1), 252 (2024). 10.1007/s40820-024-01470-w PMC11269562 39046572

[28] B.J. Neudecker, N.J. Dudney, J.B. Bates, “Lithium-free” thin-film battery with *in situ* plated Li anode. J. Electrochem. Soc. **147**(2), 517 (2000). 10.1149/1.1393226

[29] J. Qian, B.D. Adams, J. Zheng, W. Xu, W.A. Henderson et al., Anode-free rechargeable lithium metal batteries. Adv. Funct. Mater. **26**(39), 7094–7102 (2016). 10.1002/adfm.201602353

[30] L. Liu, Y. Xiang, X. Lu, J. Wang, Planar Li deposition and dissolution enable practical anode-free pouch cells. Nature **652**(8112), 1211–1218 (2026). 10.1038/s41586-026-10402-0 41845019

[31] S. Qiu, H. Zhu, S. Cheng, J. Xie, Active sodium loss in practical anode-free sodium batteries: mechanisms, challenges, and strategies. Energy Environ. Sci. **19**(9), 2788–2813 (2026). 10.1039/d6ee00760k

[32] J. Wang, Y. Zhou, Y. Zhuo, K. Fang, S. Wang et al., The challenges and strategies towards high-performance anode-free post-lithium metal batteries. Chem. Sci. **16**(2), 552–574 (2025). 10.1039/d4sc06630h PMC11633662 39669177

[33] J. Sun, Y. Du, Y. Liu, D. Yan, X. Li et al., Recent advances in potassium metal batteries: electrodes, interfaces and electrolytes. Chem. Soc. Rev. **54**(5), 2543–2594 (2025). 10.1039/d4cs00845f 39918241

[34] P. Xu, F. Huang, Y. Sun, Y. Lei, X. Cao et al., Anode-free alkali metal batteries: from laboratory to practicability. Adv. Funct. Mater. **34**(44), 2406080 (2024). 10.1002/adfm.202406080

[35] Y. An, Y. Zeng, D. Luan, X.W.D. Lou, Materials design for high-energy-density anode-free batteries. Matter **7**(4), 1466–1502 (2024). 10.1016/j.matt.2024.02.012

[36] H. Li, F. Wu, J. Wang, J. Wang, H. Qu et al., Anode-free sodium metal batteries: optimisation of electrolytes and interphases. Energy Environ. Sci. **18**(9), 3887–3916 (2025). 10.1039/d5ee00136f

[37] Y. Zhong, Q. Shi, C. Zhu, Y. Zhang, M. Li et al., Mechanistic insights into fast charging and discharging of the sodium metal battery anode: a comparison with lithium. J. Am. Chem. Soc. **143**(34), 13929–13936 (2021). 10.1021/jacs.1c06794 34410696

[38] D. Petersen, M. Gronenberg, G. Lener, E.P.M. Leiva, G.L. Luque et al., Anode-free post-Li metal batteries. Mater. Horiz. **11**(23), 5914–5945 (2024). 10.1039/d4mh00529e 39268565

[39] J. Hu, H. Wang, S. Wang, Y. Lei, L. Qin et al., Electrochemical deposition mechanism of sodium and potassium. Energy Storage Mater. **36**, 91–98 (2021). 10.1016/j.ensm.2020.12.017

[40] J. Su, C. Huang, Direct visualization and mechanistic insights into initial lithium plating in anode-free lithium metal batteries. Energy Environ. Sci. **18**(19), 8815–8826 (2025). 10.1039/d5ee01956g

[41] W.-Z. Huang, C.-Z. Zhao, P. Wu, H. Yuan, W.-E. Feng et al., Anode-free solid-state lithium batteries: a review. Adv. Energy Mater. **12**(26), 2201044 (2022). 10.1002/aenm.202201044

[42] Z. Shen, X. Jin, J. Tian, M. Li, Y. Yuan et al., Cation-doped ZnS catalysts for polysulfide conversion in lithium–sulfur batteries. Nat. Catal. **5**(6), 555–563 (2022). 10.1038/s41929-022-00804-4

[43] S.-Y. Sun, X.-Q. Zhang, X.-Y. Yan, Z. Zheng, Q.-K. Zhang et al., Advances in high-coulombic-efficiency lithium metal anodes under practical conditions in liquid electrolytes. EES Batter. **1**(3), 340–363 (2025). 10.1039/d4eb00034j

[44] S. Nanda, A. Gupta, A. Manthiram, Anode-free full cells: a pathway to high-energy density lithium-metal batteries. Adv. Energy Mater. **11**(2), 2000804 (2021). 10.1002/aenm.202000804

[45] C.-J. Huang, B. Thirumalraj, H.-C. Tao, K.N. Shitaw, H. Sutiono et al., Decoupling the origins of irreversible coulombic efficiency in anode-free lithium metal batteries. Nat. Commun. **12**, 1452 (2021). 10.1038/s41467-021-21683-6 PMC7933276 33664259

[46] M. Genovese, A.J. Louli, R. Weber, S. Hames, J.R. Dahn, Measuring the coulombic efficiency of lithium metal cycling in anode-free lithium metal batteries. J. Electrochem. Soc. **165**(14), A3321–A3325 (2018). 10.1149/2.0641814jes

[47] U.-H. Kim, G.-T. Park, B.-K. Son, G.W. Nam, J. Liu et al., Heuristic solution for achieving long-term cycle stability for Ni-rich layered cathodes at full depth of discharge. Nat. Energy **5**(11), 860–869 (2020). 10.1038/s41560-020-00693-6

[48] R. Weber, M. Genovese, A.J. Louli, S. Hames, C. Martin et al., Long cycle life and dendrite-free lithium morphology in anode-free lithium pouch cells enabled by a dual-salt liquid electrolyte. Nat. Energy **4**(8), 683–689 (2019). 10.1038/s41560-019-0428-9

[49] S. Cong, Z. Yang, X. Wan, H. Chen, S. Yuan et al., Gel electrolyte featuring parasitic salt-phobic network enables anode-free lithium batteries with long cycle life and enhanced thermal stability. Joule **10**(4), 102296 (2026). 10.1016/j.joule.2025.102296

[50] Y. An, Z. Pei, D. Luan, X.W.D. Lou, Growing sodiophilic ZnO nanorod arrays on Al substrate for high-energy-density anode-free Na batteries. J. Am. Chem. Soc. **147**(50), 46440–46448 (2025). 10.1021/jacs.5c16420 PMC12715786 41343730

[51] H. Kwon, J.-H. Lee, Y. Roh, J. Baek, D.J. Shin et al., An electron-deficient carbon current collector for anode-free Li-metal batteries. Nat. Commun. **12**, 5537 (2021). 10.1038/s41467-021-25848-1 PMC8452779 34545077

[52] M. Mao, X. Ji, Q. Wang, Z. Lin, M. Li et al., Anion-enrichment interface enables high-voltage anode-free lithium metal batteries. Nat. Commun. **14**, 1082 (2023). 10.1038/s41467-023-36853-x PMC9968319 36841872

[53] P. Liang, H. Sun, C.-L. Huang, G. Zhu, H.-C. Tai et al., A nonflammable high-voltage 4.7 V anode-free lithium battery. Adv. Mater. **34**(51), 2207361 (2022). 10.1002/adma.202207361 36193778

[54] A. Shao, H. Wang, M. Zhang, J. Liu, L. Cheng et al., Multiscale interfacial stabilization *via* prelithiation separator engineering for Ah-level anode-free lithium batteries. Nat. Commun. **16**(1), 4145 (2025). 10.1038/s41467-025-59521-8 PMC12049431 40319029

[55] S. Ma, J. Zhao, H. Xiao, Q. Gao, F. Li et al., Modulating the inner Helmholtz plane towards stable solid electrolyte interphase by anion-π interactions for high-performance anode-free lithium metal batteries. Angew. Chem. Int. Ed. **64**(1), e202412955 (2025). 10.1002/anie.202412955 39319374

[56] J. Wang, Y. Wang, X. Lu, J. Qian, C. Yang et al., Ultra-sleek high entropy alloy tights: realizing superior cyclability for anode-free battery. Adv. Mater. **36**(11), 2308257 (2024). 10.1002/adma.202308257 38102857

[57] K. Yan, Z. Lu, H.-W. Lee, F. Xiong, P.-C. Hsu et al., Selective deposition and stable encapsulation of lithium through heterogeneous seeded growth. Nat. Energy **1**, 16010 (2016). 10.1038/nenergy.2016.10

[58] D.-Y. Han, S. Kim, J. Son, S. Lee, S. Cho et al., Synergistic coupling of host and electrolyte achieving 1270 Wh L^−1^ in anode-free lithium metal batteries. Adv. Mater. **38**(14), e15906 (2026). 10.1002/adma.202515906 PMC12966975 41178147

[59] O. Tamwattana, H. Park, J. Kim, I. Hwang, G. Yoon et al., High-dielectric polymer coating for uniform lithium deposition in anode-free lithium batteries. ACS Energy Lett. **6**(12), 4416–4425 (2021). 10.1021/acsenergylett.1c02224

[60] S. Wang, Y. Wang, Z. Ouyang, S. Geng, Q. Chen et al., Molecular engineering of two-dimensional polyamide interphase layers for anode-free lithium metal batteries. Nat. Mater. **24**(12), 1957–1967 (2025). 10.1038/s41563-025-02339-y 40921748

[61] H. Yoon, D. Kim, C. Choi, J.-M. Oh, J. Shin et al., Unveiling the hidden interface: pre-SEI governs Li morphology in anode-free Li metal batteries. Adv. Mater. **38**(30), e21411 (2026). 10.1002/adma.202521411 41612614

[62] X.-L. Zhang, L. Ma, Y.-P. Cai, J. Fransaer, Q. Zheng, A low-Fermi-level current collector enables anode-free lithium metal batteries with long cycle life. Matter **7**(2), 583–602 (2024). 10.1016/j.matt.2023.11.017

[63] A.-M. Li, T.P. Pollard, Z. Wang, N. Zhang, F. Omenya et al., Non-fluorinated electrolyte for high-voltage anode-free sodium metal battery. Nat. Sustain. **9**(2), 306–316 (2026). 10.1038/s41893-025-01710-w

[64] Y.-Y. Zhang, C.-H. Zhang, Y.-J. Guo, M. Fan, Y. Zhao et al., Refined electrolyte and interfacial chemistry toward realization of high-energy anode-free rechargeable sodium batteries. J. Am. Chem. Soc. **145**(47), 25643–25652 (2023). 10.1021/jacs.3c07804 37970704

[65] Y.-Y. Zhang, C.-H. Zhang, Y.-J. Guo, X.-S. Zhang, M. Fan et al., Low-electromotive force-driven sodium compensation for optimizing Na deposition in rechargeable sodium batteries. J. Am. Chem. Soc. **147**(30), 26573–26583 (2025). 10.1021/jacs.5c06704 40674260

[66] C. Vaalma, D. Buchholz, M. Weil, S. Passerini, A cost and resource analysis of sodium-ion batteries. Nat. Rev. Mater. **3**(4), 18013 (2018). 10.1038/natrevmats.2018.13

[67] R. Usiskin, Y. Lu, J. Popovic, M. Law, P. Balaya et al., Fundamentals, status and promise of sodium-based batteries. Nat. Rev. Mater. **6**(11), 1020–1035 (2021). 10.1038/s41578-021-00324-w

[68] K.M. Abraham, How comparable are sodium-ion batteries to lithium-ion counterparts? ACS Energy Lett. **5**(11), 3544–3547 (2020). 10.1021/acsenergylett.0c02181

[69] T. Fuchs, T. Ortmann, J. Becker, C.G. Haslam, M. Ziegler et al., Imaging the microstructure of lithium and sodium metal in anode-free solid-state batteries using electron backscatter diffraction. Nat. Mater. **23**(12), 1678–1685 (2024). 10.1038/s41563-024-02006-8 PMC11599044 39313556

[70] Y. Chen, H. Li, S. Tu, J. Song, C. Ye et al., Grain-optimized copper current collectors for highly stable anode-free sodium batteries. Adv. Mater. **38**(11), e22541 (2026). 10.1002/adma.202522541 41495967

[71] S. Li, H. Zhu, Y. Liu, Q. Wu, S. Cheng et al., Space-confined guest synthesis to fabricate Sn-monodispersed N-doped mesoporous host toward anode-free Na batteries. Adv. Mater. **35**(32), 2301967 (2023). 10.1002/adma.202301967 37167932

[72] R. Zhuang, X. Zhang, C. Qu, X. Xu, J. Yang et al., Fluorinated porous frameworks enable robust anode-less sodium metal batteries. Sci. Adv. **9**(39), eadh8060 (2023). 10.1126/sciadv.adh8060 PMC11090372 37774016

[73] J. Ruan, J. Hu, Q. Li, S. Luo, J. Yang et al., Current collector interphase design for high-energy and stable anode-less sodium batteries. Nat. Sustain. **8**(5), 530–541 (2025). 10.1038/s41893-025-01545-5

[74] W. Zhang, J. Zheng, Z. Ren, J. Wang, J. Luo et al., Anode-free sodium metal pouch cell using Cu_3_P nanowires *in situ* grown on current collector. Adv. Mater. **36**(15), 2310347 (2024). 10.1002/adma.202310347 38174663

[75] Y. An, Z. Pei, D. Luan, X.W.D. Lou, A functionalized porous Al current collector enables high-energy density anode-free Na batteries. Sci. Adv. **11**(34), eadx7124 (2025). 10.1126/sciadv.adx7124 PMC12366698 40834082

[76] H. Zhu, L. Peng, J. Wu, S. Li, Q. Wu et al., Fluorine-doped micropore-covered mesoporous carbon nanofibers for long-lasting anode-free sodium metal batteries. Nat. Commun. **16**(1), 5494 (2025). 10.1038/s41467-025-60168-8 PMC12216415 40593488

[77] Y. An, Z. Pei, D. Luan, X.W.D. Lou, Foldable anode-free sodium batteries enabled by N, P-codoped carbon macroporous fibers incorporated with CoP nanoparticles. Sci. Adv. **11**(19), eadv2007 (2025). 10.1126/sciadv.adv2007 PMC12063667 40344062

[78] Z. Hu, L. Liu, X. Wang, H. Lu, Q. Zheng et al., *In situ* integration of rapid ion-diffusion interlayers on Cu current collectors toward ultrafast anode-free sodium metal batteries. ACS Nano **19**(25), 23193–23208 (2025). 10.1021/acsnano.5c05043 40519174

[79] Z. Wang, R. Tian, H. Jiang, G. Chen, Z. Shen et al., A k descriptor to design of current collectors for anode-free sodium batteries. Adv. Mater. **37**(40), 2504760 (2025). 10.1002/adma.202504760 40696969

[80] W. Zhang, Y. Liu, Z. Guo, Approaching high-performance potassium-ion batteries *via* advanced design strategies and engineering. Sci. Adv. **5**(5), eaav7412 (2019). 10.1126/sciadv.aav7412 PMC6510555 31093528

[81] B. Ji, W. Yao, Y. Zheng, P. Kidkhunthod, X. Zhou et al., A fluoroxalate cathode material for potassium-ion batteries with ultra-long cyclability. Nat. Commun. **11**(1), 1225 (2020). 10.1038/s41467-020-15044-y PMC7060185 32144250

[82] H. Ding, J. Wang, J. Zhou, C. Wang, B. Lu, Building electrode skins for ultra-stable potassium metal batteries. Nat. Commun. **14**(1), 2305 (2023). 10.1038/s41467-023-38065-9 PMC10121571 37085541

[83] Y. Zhao, B. Liu, Y. Yi, X. Lian, M. Wang et al., An anode-free potassium-metal battery enabled by a directly grown graphene-modulated aluminum current collector. Adv. Mater. **34**(29), 2202902 (2022). 10.1002/adma.202202902 35584284

[84] Z. Yu, Q. Liu, D. Wang, J. Shi, D. Zhai et al., Unraveling electrode surface chemistry in determining interphase stability and deposition homogeneity for anode-free potassium metal batteries. Angew. Chem. Int. Ed. **64**(22), e202502091 (2025). 10.1002/anie.202502091 40113580

[85] Z. Cui, J. Song, M. Chen, W. Wang, W. Zhang et al., Non-completely selenized Cu-OSe nanowires as potassium-philic host for anode-free potassium metal batteries. Energy Storage Mater. **71**, 103649 (2024). 10.1016/j.ensm.2024.103649

[86] J. Sun, S. Zhang, J. Li, B. Xie, J. Ma et al., Robust transport: an artificial solid electrolyte interphase design for anode-free lithium-metal batteries. Adv. Mater. **35**(20), 2209404 (2023). 10.1002/adma.202209404 36573477

[87] Z. Ouyang, S. Wang, Y. Wang, S. Muqaddas, S. Geng et al., An ultralight composite current collector enabling high-energy-density and high-rate anode-free lithium metal battery. Adv. Mater. **36**(33), 2407648 (2024). 10.1002/adma.202407648 38900369

[88] Y. Wang, Z. Qu, S. Geng, M. Liao, L. Ye et al., Anode-free lithium metal batteries based on an ultrathin and respirable interphase layer. Angew. Chem. Int. Ed. **62**(27), e202304978 (2023). 10.1002/anie.202304978 37139890

[89] S.T. Oyakhire, W. Zhang, A. Shin, R. Xu, D.T. Boyle et al., Electrical resistance of the current collector controls lithium morphology. Nat. Commun. **13**, 3986 (2022). 10.1038/s41467-022-31507-w PMC9276694 35821247

[90] J. Pu, J. Li, K. Zhang, T. Zhang, C. Li et al., Conductivity and lithiophilicity gradients guide lithium deposition to mitigate short circuits. Nat. Commun. **10**(1), 1896 (2019). 10.1038/s41467-019-09932-1 PMC6478682 31015466

[91] S. Li, H. Zhu, Y. Liu, Z. Han, L. Peng et al., Codoped porous carbon nanofibres as a potassium metal host for nonaqueous K-ion batteries. Nat. Commun. **13**(1), 4911 (2022). 10.1038/s41467-022-32660-y PMC9392754 35987982

[92] C.-P. Yang, Y.-X. Yin, S.-F. Zhang, N.-W. Li, Y.-G. Guo, Accommodating lithium into 3D current collectors with a submicron skeleton towards long-life lithium metal anodes. Nat. Commun. **6**, 8058 (2015). 10.1038/ncomms9058 PMC4560781 26299379

[93] C. Wang, M. Liu, M. Thijs, F.G.B. Ooms, S. Ganapathy et al., High dielectric barium titanate porous scaffold for efficient Li metal cycling in anode-free cells. Nat. Commun. **12**(1), 6536 (2021). 10.1038/s41467-021-26859-8 PMC8585873 34764287

[94] H. Zhao, D. Lei, Y.-B. He, Y. Yuan, Q. Yun et al., Compact 3D copper with uniform porous structure derived by electrochemical dealloying as dendrite-free lithium metal anode current collector. Adv. Energy Mater. **8**(19), 1800266 (2018). 10.1002/aenm.201800266

[95] M.-H. Kim, D.Y. Kim, Y. Li, J. Kim, M.H. Kim et al., Horizontal lithium growth driven by surface dynamics on single crystal Cu(111) foil. Energy Environ. Sci. **17**(18), 6521–6532 (2024). 10.1039/d4ee01766h

[96] X. Cao, X. Ren, L. Zou, M.H. Engelhard, W. Huang et al., Monolithic solid–electrolyte interphases formed in fluorinated orthoformate-based electrolytes minimize Li depletion and pulverization. Nat. Energy **4**(9), 796–805 (2019). 10.1038/s41560-019-0464-5

[97] S. Ko, T. Obukata, T. Shimada, N. Takenaka, M. Nakayama et al., Electrode potential influences the reversibility of lithium-metal anodes. Nat. Energy **7**(12), 1217–1224 (2022). 10.1038/s41560-022-01144-0

[98] Q.-K. Zhang, X.-Q. Zhang, J. Wan, N. Yao, T.-L. Song et al., Homogeneous and mechanically stable solid–electrolyte interphase enabled by trioxane-modulated electrolytes for lithium metal batteries. Nat. Energy **8**(7), 725–735 (2023). 10.1038/s41560-023-01275-y

[99] M. Abdollahifar, A. Paolella, Anode-free batteries: the energy density prize and the stability paradox. ACS Energy Lett. **11**(2), 1323–1348 (2026). 10.1021/acsenergylett.5c03401

[100] P. Poizot, S. Laruelle, S. Grugeon, L. Dupont, J.-M. Tarascon, Nano-sized transition-metal oxides as negative-electrode materials for lithium-ion batteries. Nature **407**(6803), 496–499 (2000). 10.1038/35035045 11028997

[101] Y. Lu, L. Yu, X.W.D. Lou, Nanostructured conversion-type anode materials for advanced lithium-ion batteries. Chem **4**(5), 972–996 (2018). 10.1016/j.chempr.2018.01.003

[102] K. He, F. Lin, Y. Zhu, X. Yu, J. Li et al., Sodiation kinetics of metal oxide conversion electrodes: a comparative study with lithiation. Nano Lett. **15**(9), 5755–5763 (2015). 10.1021/acs.nanolett.5b01709 26288360

[103] K. Tang, L. Tian, Y. Zhang, Z.J. Xu, Anode-free lithium metal batteries: a promising flexible energy storage system. J. Mater. Chem. A **12**(27), 16268–16292 (2024). 10.1039/d4ta02003k

[104] C. Fang, B. Lu, G. Pawar, M. Zhang, D. Cheng et al., Pressure-tailored lithium deposition and dissolution in lithium metal batteries. Nat. Energy **6**(10), 987–994 (2021). 10.1038/s41560-021-00917-3

[105] X. Zhang, Q.J. Wang, K.L. Harrison, K. Jungjohann, B.L. Boyce et al., Rethinking how external pressure can suppress dendrites in lithium metal batteries. J. Electrochem. Soc. **166**(15), A3639–A3652 (2019). 10.1149/2.0701914jes

[106] Y. Ren, W. Shin, A. Manthiram, Operating high-energy lithium-metal pouch cells with reduced stack pressure through a rational lithium-host design. Adv. Energy Mater. **12**(19), 2200190 (2022). 10.1002/aenm.202200190

[107] A.J. Louli, A. Eldesoky, R. Weber, M. Genovese, M. Coon et al., Diagnosing and correcting anode-free cell failure *via* electrolyte and morphological analysis. Nat. Energy **5**(9), 693–702 (2020). 10.1038/s41560-020-0668-8

[108] S. Moradi, B. Zahiri, P.V. Braun, Cathode chemomechanics controls Li metal solid-state battery performance under low stack pressures. Nat. Commun. **16**(1), 9266 (2025). 10.1038/s41467-025-64358-2 PMC12537972 41115896

[109] K. Tang, L. Tian, Z. Shen, W. Xie, S.K.J. Lim et al., Seeding a gradient solid-electrolyte interphase in anode-free lithium metal batteries. Nano Lett. **26**(4), 1211–1219 (2026). 10.1021/acs.nanolett.5c04732 41586505

[110] M. Burton, S. Narayanan, B. Jagger, L.F. Olbrich, S. Dhir et al., Techno-economic assessment of thin lithium metal anodes for solid-state batteries. Nat. Energy **10**(1), 135–147 (2025). 10.1038/s41560-024-01676-7

[111] N. Kittner, F. Lill, D.M. Kammen, Energy storage deployment and innovation for the clean energy transition. Nat. Energy **2**, 17125 (2017). 10.1038/nenergy.2017.125

[112] O. Schmidt, A. Hawkes, A. Gambhir, I. Staffell, The future cost of electrical energy storage based on experience rates. Nat. Energy **2**, 17110 (2017). 10.1038/nenergy.2017.110

[113] A. Yao, S.M. Benson, W.C. Chueh, Critically assessing sodium-ion technology roadmaps and scenarios for techno-economic competitiveness against lithium-ion batteries. Nat. Energy **10**(3), 404–416 (2025). 10.1038/s41560-024-01701-9

[114] J.T. Frith, M.J. Lacey, U. Ulissi, A non-academic perspective on the future of lithium-based batteries. Nat. Commun. **14**(1), 420 (2023). 10.1038/s41467-023-35933-2 PMC9879955 36702830

[115] P.M. Attia, A. Grover, N. Jin, K.A. Severson, T.M. Markov et al., Closed-loop optimization of fast-charging protocols for batteries with machine learning. Nature **578**(7795), 397–402 (2020). 10.1038/s41586-020-1994-5 32076218

[116] K.A. Severson, P.M. Attia, N. Jin, N. Perkins, B. Jiang et al., Data-driven prediction of battery cycle life before capacity degradation. Nat. Energy **4**(5), 383–391 (2019). 10.1038/s41560-019-0356-8

[117] P. Ma, R. Kumar, K.-H. Wang, C.V. Amanchukwu, Active learning accelerates electrolyte solvent screening for anode-free lithium metal batteries. Nat. Commun. **16**, 8396 (2025). 10.1038/s41467-025-63303-7 PMC12462480 40998777

[118] S.C. Kim, S.T. Oyakhire, C. Athanitis, J. Wang, Z. Zhang et al., Data-driven electrolyte design for lithium metal anodes. Proc. Natl. Acad. Sci. **120**(10), e2214357120 (2023). 10.1073/pnas.2214357120 PMC10013853 36848560

